# 2022 CSACI annual scientific meeting book of abstracts

**DOI:** 10.1186/s13223-024-00898-4

**Published:** 2024-08-06

## Allergic rhinitis/asthma

## 1 Efficacy of tezepelumab in patients with severe, uncontrolled asthma and perennial aeroallergen sensitization: a pooled analysis of the phase 2b PATHWAY and phase 3 NAVIGATOR studies

### Andrew Menzies-Gow^1^, Jonathan Corren^2^, Christopher S. Ambrose^3^, Bill Cook^3^, Åsa Hellqvist^4^, Stephanie L. Roseti^5^, Neil Martin^6,7^, Andrew W. Lindsley^8^, Gene Colice^5^

#### ^1^Royal Brompton and Harefield Hospitals, London, United Kingdom; ^2^David Geffen School of Medicine, UCLA, Los Angeles, CA, USA; ^3^Respiratory and Immunology, BioPharmaceuticals Medical, AstraZeneca, Gaithersburg, MD, USA; ^4^Biometrics, Late-stage Development, Respiratory and Immunology, BioPharmaceuticals R&D, AstraZeneca, Gothenburg, Sweden; ^5^Late-stage Development, Respiratory and Immunology, BioPharmaceuticals R&D, AstraZeneca, Gaithersburg, MD, USA; ^6^Respiratory and Immunology, BioPharmaceuticals Medical, AstraZeneca, Cambridge, United Kingdom; ^7^Honorary Senior Lecturer, University of Leicester, Leicester, United Kingdom; ^8^US Medical Affairs, Amgen, Thousand Oaks, CA, USA

##### **Correspondence:** Bill Cook

**Allergy, Asthma & Clinical Immunology** 2024, **20(Suppl 2)**:1

**Background:** Allergic asthma is present in approximately 60% of adult patients with severe asthma. Tezepelumab, a human monoclonal antibody, blocks thymic stromal lymphopoietin (TSLP). This post hoc analysis evaluated the efficacy of tezepelumab with increased statistical precision in patients with severe, uncontrolled asthma and perennial aeroallergen sensitization using pooled data from the phase 2b PATHWAY and phase 3 NAVIGATOR studies.

**Methods:** PATHWAY (NCT02054130) and NAVIGATOR (NCT03347279) were multicentre, randomized, double-blind, placebo-controlled studies with similar designs. Patients who received tezepelumab 210 mg or placebo subcutaneously every 4 weeks for 52 weeks and had a positive or negative fluorescence enzyme immunoassay test for serum-specific immunoglobulin E against at least one perennial aeroallergen were included in this analysis. The annualized asthma exacerbation rate (AAER) over 52 weeks (primary endpoint in both trials) and the annualized rate of exacerbations that required hospitalization or an emergency department (ED) visit over 52 weeks were assessed in patients grouped by perennial allergic status.

**Results:** Overall, 1299 patients were included; 815 (63%) had perennial aeroallergen sensitization and 484 (37%) did not. Tezepelumab reduced the AAER over 52 weeks compared with placebo by 62% (95% CI: 53–70) in patients with perennial aeroallergen sensitization and by 54% (95% CI: 38–66) in those without. The annualized rate of exacerbations that required hospitalization or an ED visit was reduced with tezepelumab compared with placebo by 80% (95% CI: 61–90) in patients with perennial aeroallergen sensitization and by 74% (95% CI: 41–88) in those without.

**Conclusions:** Tezepelumab reduced exacerbations compared with placebo, including those that required hospitalization or an ED visit, in patients with severe, uncontrolled asthma with or without perennial aeroallergen sensitization. These findings further support the benefits of tezepelumab in a broad population of patients with severe, uncontrolled asthma, including those with allergic and non-allergic asthma.

## 2 Effect of tezepelumab on seasonal exacerbations in patients with severe, uncontrolled asthma grouped by blood eosinophil count

### Jonathan Corren^1^, Andrew Menzies-Gow^2^, Christopher S. Ambrose^3^, Gene Colice^4^, Stephanie L. Roseti^4^, Asa Hellqvist^5^, Kamil Kmita^6^, Andrew W. Lindsley^7^, Bill Cook^3^

#### ^1^David Geffen School of Medicine, UCLA, Los Angeles, CA, USA; ^2^Royal Brompton and Harefield Hospitals, London, United Kingdom; ^3^Respiratory and Immunology, BioPharmaceuticals Medical, AstraZeneca, Gaithersburg, MD, USA; ^4^Late-stage Development, Respiratory and Immunology, BioPharmaceuticals R&D, AstraZeneca, Gaithersburg, MD, USA; ^5^Biometrics, Late-stage Development, Respiratory and Immunology, BioPharmaceuticals R&D, AstraZeneca, Gothenburg, Sweden; ^6^Biometrics, Late-stage Development, Respiratory and Immunology BioPharmaceuticals R&D, AstraZeneca, Warsaw, Poland; ^7^US Medical Affairs, Amgen, Thousand Oaks, CA, USA

##### **Correspondence:** Bill Cook

**Allergy, Asthma & Clinical Immunology** 2024, **20(Suppl 2)**:2

**Background:** Tezepelumab, a human monoclonal antibody, targets thymic stromal lymphopoietin (TSLP). In the phase 3 NAVIGATOR study (NCT03347279), tezepelumab reduced exacerbations in patients with severe, uncontrolled asthma with high or low baseline blood eosinophil counts (BECs) and across all seasons in the overall population. This pre-specified exploratory analysis evaluated the effect of tezepelumab on seasonal asthma exacerbation rates in NAVIGATOR patients grouped by baseline BEC.

**Methods:** NAVIGATOR was a multicentre, randomized, double-blind, placebo-controlled study. Patients (12–80 years old) were randomized 1:1 to tezepelumab 210 mg or placebo subcutaneously every 4 weeks for 52 weeks. The annualized asthma exacerbation rate (AAER) was assessed by season in patients grouped by baseline BEC. Data from patients in the southern hemisphere were transformed to align with northern hemisphere seasons.

**Results:** Of 1059 treated patients, 618 had a BEC of < 300 cells/μL and 441 had a BEC of ≥ 300 cells/μL at baseline. In the placebo group, there were seasonal variations in the AAER, with a peak in winter of 2.32 (95% confidence interval [CI]: 1.85, 2.91) and 3.07 (95% CI: 2.36, 4.00) in patients with BEC of < 300 cells/µL and ≥ 300 cells/μL, respectively. In patients with a BEC of < 300 cells/μL, tezepelumab reduced the AAER versus placebo by 31% (95% CI: − 4, 54) in spring, 37% (95% CI: 5, 58) in summer, 43% (95% CI: 20, 59) in fall and 55% (95% CI: 36, 68) in winter. In patients with a BEC of ≥ 300 cells/μL, tezepelumab reduced the AAER versus placebo by 62% (95% CI: 38, 77) in spring, 80% (95% CI: 67, 88) in summer, 66% (95% CI: 49, 77) in fall and 72% (95% CI: 57, 82) in winter.

**Conclusions:** Tezepelumab reduced exacerbations versus placebo across all seasons in patients with severe, uncontrolled asthma with high or low baseline BECs, consistent with the overall NAVIGATOR population.

## 3 Analysing phenotypes post exposure in allergic rhinitis (APPEAR): comparing phenotype distribution in the environmental exposure unit and nasal allergen challenge

### Abigail Davis^1,2^, Sophia Linton^1,2,3^, Jenny Thiele^1,2^, Lubnaa Hossenbaccus^1,2,3^, Hannah Botting^2^, Terry Walker^2^, Lisa Steacy^2^, Anne K. Ellis^1,2,3^

#### ^1^Department of Biomedical and Molecular Sciences, Queen’s University, Kingston, ON; ^2^Allergy Research Unit, Kingston Health Sciences Center—KGH Site, Kingston, ON, ^3^Department of Medicine, Queen’s University, Kingston, ON

##### **Correspondence:** Abigail Davis

**Allergy, Asthma & Clinical Immunology** 2024, **20(Suppl 2)**:3

**Background:** Previous studies described clinical phenotypes of allergic rhinitis (AR) based on total nasal symptom score (TNSS) as early-phase responders (EPRs), protracted early-phase responders (pEPRs), and dual responders (DRs) [1–3]. The purpose of APPEAR was to determine if these phenotypes could be validated using different allergens (birch, grass, ragweed, and house dust mite (HDM)), and different AR models (nasal allergen challenge (NAC), and Environmental Exposure Unit (EEU)).

**Methods:** The APPEAR database consisted of 252 allergen exposures from NAC (n = 7) and EEU (n = 4) studies ranging from 2010 to 2021. Participants were phenotyped based on criteria described previously [1]. Statistical analyses were performed on GraphPad Prism 9.0.

**Results:** The phenotypes described by Soliman & Ellis (2015) were present across all models and allergens. A novel low responder (LoR) phenotype was defined; participants experienced dampened symptomatic response (TNSS < 4). EPRs accounted for 124 (49.2%) participants followed by 77 (30.6%) pEPRs, 32 (12.7%) DRs, and 19 (7.5%) LoRs. Phenotype distribution between NAC versus EEU and allergens did not differ significantly. A strong negative correlation between TNSS and peak nasal inspiratory flow (PNIF) existed when grouping by phenotype (EPR, R^2^ = 0.82, p < 0.0001; pEPR, R^2^ = 0.78, p < 0.0001; DR, R^2^ = 0.78; LoR, R^2^ = 0.83, p < 0.0001).

**Conclusions:** AR phenotypes were reproducible in this larger sample size amongst all allergens and models used. We demonstrated that the same AR phenotypes can be identified using subjective TNSS and/or objective PNIF scoring methods. We established participants likely exhibit their intrinsic phenotypes regardless of the allergen exposure model or allergen used. Further research is warranted to investigate differences between perennial and seasonal allergens due to limited number of participants in HDM NAC. Future studies should be designed to directly compare the NAC versus EEU model in the same participant.

**References**

1. Soliman M, Ellis AK. Phenotyping allergic rhinitis as early- or dual-phase responses using the environmental exposure unit. Ann Allergy Asthma Immunol. 2015; 114:344–345.
2. Rawls M, Thiele J, Adams DE, Steacy LM, Ellis AK. Clinical symptoms and biomarkers of Bermuda grass–induced allergic rhinitis using the nasal allergen challenge model. Ann Allergy Asthma Immunol. 2020; 124:608-615.e2.
3. Hossenbaccus L, Linton S, Thiele J, Steacy L, Walker T, Malone C, et al. Clinical validation of controlled exposure to house dust mite in the environmental exposure unit (EEU). Allergy Asthma Clin Immunol. 2021; 17:34.

## 4 Survey of Canadian prescribers of subcutaneous allergy immunotherapy

### Daniel H. Li^1^, Harold Kim^2,3^, Stephen Betschel^1^

#### ^1^University of Toronto, Toronto, ON; ^2^Western University, London, ON; ^3^McMaster University, Hamilton, ON

##### **Correspondence:** Daniel H. Li

**Allergy, Asthma & Clinical Immunology** 2024, **20(Suppl 2)**:4

**Background:** Subcutaneous immunotherapy (SCIT) is a standard approach used by allergists to treat patients with aeroallergen sensitization. However, administration errors in the delivery of SCIT can lead to serious systemic reactions. Quality improvement projects are important to try to improve the safety of this process and a key first step would be to identify where to implement these interventions. Given that there is no Canadian data which identifies the location of where SCIT is administered and the satisfaction of instructions provided to practitioners to administer SCIT, the first step was to determine this by survey methodology.

**Methods:** A brief seven question survey was generated to examine different facets of SCIT administration including: the number of patients per allergist per year typically started on SCIT, where patients receive their SCIT injections, who performs the injections, satisfaction with the instructions provided to administer SCIT and the geographic distribution of respondents to ensure adequate representation across Canada. The survey was sent out via the Canadian Society of Allergy and Clinical Immunology (CSACI) April 2022 newsletter addressed to practicing allergists in Canada.

**Results:** A total of 35 responses were received for our survey with diverse representation of each region across Canada. Most allergists initiate less than 100 patients per year on SCIT for aeroallergens. 75% of respondents have 50% to 75% of their patients on SCIT receive injections at their family physicians’ offices. For those that administer SCIT in their clinic, it is typically done by the clinic nurse 50% of the time. 80% of respondents were satisfied with the instructions provided by manufacturers with their SCIT.

**Conclusions:** Most patients receive their SCIT injections at their family physician’s office. Future surveys to identify key areas of quality improvement regarding the safety of SCIT administration will need to focus on this setting.

## 5 Biomarkers associated with lung function decline and dupilumab response in patients with moderate-to-severe asthma: LIBERTY ASTHMA QUEST study

### Ian D. Pavord^1^, Guy Brusselle^2^, Daniel J. Jackson^3^, Christopher E. Brightling^4^, Alberto Papi^5^, Jorge F. Maspero^6^, Klaus F. Rabe^7,8^, Stephanie Korn^9^, Mei Zhang^10^, Nami Pandit-Abid^10^, Sven Sorensen^11^, Megan Hardin^12^, Lucia De Prado Gómez^13^, Juby A. Jacob-Nara^10^, Paul Rowe^10^

#### ^1^NIHR Oxford Biomedical Research Centre, University of Oxford, Oxford, United Kingdom; ^2^Ghent University, Ghent, Belgium; ^3^University of Wisconsin School of Medicine and Public Health, Madison, WI, USA; ^4^University of Leicester, Leicester, United Kingdom; ^5^Respiratory Medicine Unit, University of Ferrara, S. Anna University Hospital, Ferrara, Italy; ^6^Fundación CIDEA, Buenos Aires, Argentina; ^7^LungenClinic Grosshansdorf (member of the German Center for Lung Research [DZL]), Airway Research Center North (ARCN), Grosshansdorf, Germany; ^8^Christian-Albrechts University (member of the German Center for Lung Research [DZL]), Airway Research Center North (ARCN), Kiel, Germany; ^9^IKF Pneumologie Mainz and Thoraxklinik, Heidelberg, Germany; ^10^Sanofi, Bridgewater, NJ, USA; ^11^Sanofi, Mississauga, ON, ^12^Sanofi, Cambridge, MA, USA, ^13^Sanofi, Reading, United Kingdom

##### **Correspondence:** Sven Sorensen

**Allergy, Asthma & Clinical Immunology** 2024, **20(Suppl 2)**:5

**Background:** Patients with moderate-to-severe asthma may be at risk of accelerated lung function decline (LFD). Currently, there are limited data on prognostic and predictive biomarkers for LFD. Dupilumab, a human monoclonal antibody, blocks interleukin 4/13 signaling, key and central drivers of type 2 inflammation. In phase 3 QUEST (NCT02414854), dupilumab improved lung function and was generally well tolerated. Here we assessed baseline characteristics associated with rapid LFD in patients who received placebo during QUEST and the effect of dupilumab on LFD.

**Methods:** Baseline characteristics were assessed in patients who received placebo during QUEST with rapid (> 2%/year) or no (< 1%/year) LFD (defined as post-bronchodilator FEV1 percent change). To evaluate the predictive/prognostic role of biomarkers, LFD was evaluated across different baseline blood eosinophil (eos) (< 150, ≥ 150, ≥ 300 cells/mL) and FeNO (< 25, ≥ 25, ≥ 50 ppb) thresholds across dupilumab and placebo populations.

**Results:** Placebo patients with rapid LFD had higher baseline FeNO levels (36 ppb, n = 271) compared with patients without LFD (27 ppb, n = 49), but similar eos. Placebo patients with elevated baseline FeNO had greater lung function decline, irrespective of baseline eos: LFD in placebo patients with high baseline FeNO ≥ 25 and ≥ 50 combined with low eos < 150 was − 116 mL/year (n = 53) and − 102 mL/year (n = 20), respectively, compared to − 56 mL/year (n = 187) and -71 mL/year (n = 90) in patients with low FeNO < 25 and high eos ≥ 150 and EOS ≥ 300, respectively. LFD was attenuated in dupilumab-treated patients across populations, ranging from + 43 (n = 27) to + 4 (n = 99) in the high FeNO/low eos group and from − 17 (n = 363) to − 35 (n = 172) in the low FeNO/high eos group.

**Conclusions:** FeNO was elevated in patients with rapid LFD vs no LFD. High baseline FeNO levels were associated with greater LFD in placebo patients, which was attenuated in dupilumab-treated patients, suggesting FeNO may be prognostic for accelerated LFD and predictive of the treatment response to dupilumab.

## 6 The effect of allergen sensitization status on dupilumab efficacy on lung function in VOYAGE pediatric patients with uncontrolled, moderate-to-severe type 2 inflammatory asthma

### Wanda Phipatanakul^2,3^, Nikolaos G. Papadopoulos^4,5^, Vivian Hernandez-Trujillo^6^, Eckard Hamelmann^7^, Robert S. Zeiger^8^, Francine M. Ducharme^9^, Neal Jain^10^, Changming Xia^11^, Sven Sorensen^1^, Rebecca Gall^11^, Nami Pandit-Abid^12^, Juby A. Jacob-Nara^12^, Paul J. Rowe^12^, Yamo Deniz^11^

#### ^1^Sanofi, Mississauga, ON; ^2^Department of Allergy and Immunology, Boston Children’s Hospital, Boston, MA, USA; ^3^Department of Pediatrics, Harvard Medical School, Boston, MA, USA; ^4^Royal Manchester Children’s Hospital, Manchester, United Kingdom; ^5^2nd Pediatric Clinic, University of Athens, Athens, Greece; ^6^Allergy and Immunology Care Center of South Florida, Miami Lakes, FL, USA; ^7^Children’s Center Bethel, University of Bielefeld, Bielefeld, Germany; ^8^Kaiser Permanente Bernard J. Tyson School of Medicine, Pasadena, CA, USA; ^9^University of Montreal, Montreal, QC; ^10^Arizona Allergy and Immunology Research, Gilbert, AZ, USA; ^11^Regeneron Pharmaceuticals, Inc., Tarrytown, NY, USA; ^12^Sanofi, Bridgewater, NJ, USA

##### **Correspondence:** Sven Sorensen

**Allergy, Asthma & Clinical Immunology** 2024, **20(Suppl 2)**:6

**Background:** 85% of children with uncontrolled, moderate-to-severe asthma have type 2 (T2) inflammatory asthma (baseline blood eosinophils ≥ 150 cells/µL or FeNO ≥ 20 ppb); most of these children exhibit an allergic phenotype. Asthma can lead to severe and/or frequent exacerbations and abnormal lung function. Dupilumab (DPL), a fully human monoclonal antibody, blocks the shared receptor component for IL-4 and IL-13, key and central drivers of T2 inflammation in multiple diseases. In phase 3 VOYAGE (NCT02948959), add-on DPL vs placebo reduced severe asthma exacerbations and improved lung function in children aged 6 to 11 years with uncontrolled, moderate‑to‑severe T2 asthma. Dupilumab has demonstrated an acceptable safety profile. To determine the effect of allergen sensitization, we conducted a post hoc analysis comparing efficacy of DPL vs placebo in non-, monoallergen- and multiallergen-sensitized VOYAGE patients with T2 asthma.

**Methods:** Children were classified as non- (total serum IgE < 30 IU/mL or no perennial aeroallergen-specific IgE ≥ 0.35 kU/L at baseline), monoallergen- or multiallergen-sensitized (total baseline serum IgE ≥ 30 IU/mL in both, and 1 (mono-) or > 1 (multi-) aeroallergen-specific IgE ≥ 0.35 kU/L, respectively). The change from baseline in pre-bronchodilator (BD) percent predicted forced expiratory volume in 1 s (pre-BD FEV_1_pp) was compared.

**Results:** DPL vs placebo significantly improved pre-BD FEV_1_pp in multiallergen-sensitized patients at Weeks 2 (LSMD [95% CI]: 5.3 percentage points [1.4–9.2]; *P* = 0.007), 24 (8.7 percentage points [4.1–13.3]; *P* < 0.001) and 52 (9.0 percentage points [4.0–14.1]; *P* < 0.001) and monoallergen-sensitized patients at Weeks 24 (LSMD [95% CI]: 8.1 percentage points [2.0–14.2]; *P* = 0.01) and 52 (10.1 percentage points [4.2–16.1]; *P* = 0.001).

**Conclusions:** DPL significantly improved lung function by Week 24 in monoallergen-sensitized patients and by Week 2 in multiallergen-sensitized patients and sustained these improvements through Week 52.

## 7 Dupilumab improves exacerbations and lung function irrespective of prior asthma exacerbations: liberty asthma traverse

### Alberto Papi^1^, Mario Castro^2^, William W. Busse^3^, David Langton^4^, Stephanie Korn^5^, Changming Xia^6^, Sven Sorensen^7^, Xavier Soler^6^, Nami Pandit-Abid^8^, Juby A. Jacob-Nara^8^, Paul J. Rowe^8^, Yamo Deniz^6^

#### ^1^Respiratory Medicine Unit, University of Ferrara, S. Anna University Hospital, Ferrara, Italy; ^2^University of Kansas School of Medicine, Kansas City, KS, USA; ^3^UW Allergy, Pulmonary and Critical Care Medicine, University of Wisconsin School of Medicine and Public Health, Madison, WI, USA; ^4^Frankston Hospital, Frankston, VIC, Australia; ^5^IKF Pneumologie Mainz and Thoraxklinik Heidelberg, Mainz, Germany, ^6^Regeneron Pharmaceuticals, Inc., Tarrytown, NY, USA; ^7^Sanofi, Mississauga, ON; ^8^Sanofi, Bridgewater, NJ, USA

##### **Correspondence:** Sven Sorensen

**Allergy, Asthma & Clinical Immunology** 2024, **20(Suppl 2)**:7

**Background:** Prior asthma exacerbations have been associated with lung function decline and increased risk of future exacerbations. Dupilumab, a human monoclonal antibody, blocks the shared receptor component for interleukins 4/13, key and central drivers of type 2 inflammation in multiple diseases. In phase 3 QUEST (NCT02414854), add-on dupilumab significantly reduced exacerbations and improved lung function in patients with uncontrolled, moderate-to-severe asthma. The TRAVERSE open-label extension study (NCT02134028) evaluated dupilumab long-term safety, tolerability, and efficacy in patients from a previous dupilumab asthma study. Dupilumab safety during TRAVERSE was consistent with the known safety profile. This post hoc analysis examined the relationship between prior exacerbations and lung function in patients with type 2 asthma (blood eosinophils ≥ 150cells/μL or FeNO ≥ 25 ppb at baseline) from QUEST enrolled in TRAVERSE.

**Methods:** Patients who received placebo or dupilumab in QUEST received dupilumab 300 mg every 2 weeks in TRAVERSE for up to 48/96 weeks (placebo/dupilumab and dupilumab/dupilumab groups). This analysis assessed annualized severe exacerbation rates (AER) and change from baseline in % predicted (pp) FEV_1_ in non-exacerbators (0 exacerbations) and exacerbators (≥ 1 exacerbations) during QUEST.

**Results:** In the exacerbator group, dupilumab reduced AER vs placebo in QUEST (1.67 vs 2.59, respectively); AER was low in TRAVERSE (dupilumab/dupilumab 0.78, placebo/dupilumab 0.56). In the non-exacerbator group, dupilumab maintained low AER (dupilumab/dupilumab 0.11, placebo/dupilumab 0.17) in TRAVERSE. Mean ppFEV_1_ change at Week 2/48 was 9.08/9.88 and 11.28/13.58 for dupilumab/dupilumab and placebo/dupilumab in the exacerbator group and 13.86/14.52 and 11.59/12.88 for dupilumab/dupilumab and placebo/dupilumab in the non-exacerbator group. Improvements in lung function were maintained up to Week 96 of TRAVERSE.

**Conclusions:** Dupilumab reduced exacerbations and improved lung function in the placebo/dupilumab group and sustained improvements in the dupilumab/dupilumab group irrespective of prior exacerbation status.

## Allied health

## 8 Asthma exacerbations after Covid-19 diagnosis among children receiving asthma education

### Lenore Johnstone^2^, Nancy Ross^2^, Elinor Simons^1,2,3^

#### ^1^Section of Allergy and Clinical Immunology, Department of Pediatrics & Child Health, University of Manitoba, Winnipeg, MB; ^2^The Children’s Allergy and Asthma Education Centre (CAAEC), Winnipeg, MB, ^3^Children’s Hospital Research Institute of Manitoba, Winnipeg, MB

##### **Correspondence:** Lenore Johnstone

**Allergy, Asthma & Clinical Immunology** 2024, **20(Suppl 2)**:8

**Background:** Asthma education has been a critical support to children and families managing new and pre-existing asthma during the COVID-19 Pandemic. Much remains unknown about the relationship between COVID-19 and asthma in children. Nurse educators at the Children’s Allergy and Asthma Education Centre (CAAEC) noticed asthma exacerbations requiring education 2–3 months after a child’s diagnosis of COVID-19, and hypothesized a possible association between COVID-19 diagnosis and presentation of asthma several months later. We are evaluating this question using records of CAAEC Asthma Education.

**Methods:** We examined sequential COVID-19 positive children who received their first Asthma Education session with the CAAEC Nurse Educators from September 2021 to May 2022. We characterized the timing of their positive test for COVID-19, testing for other respiratory viruses, timing of asthma exacerbations, first or repeat presentation for asthma, and sociodemographic factors.

**Results:** Among 23 sequential children who had a reported or documented positive test for COVID-19 at the time of their asthma education with a CAAEC Nurse Educator, the median age was 3 years (range 1–15 years); 13% of children were newly diagnosed with asthma at this presentation and 22% received their education during an inpatient admission to the Children’s Hospital. The median time between positive testing for COVID-19 and asthma exacerbation was 2 months (range 0–6 months). Evaluation of documented infection with other respiratory viruses and sociodemographic features is ongoing.

**Conclusions:** CAAEC Nurse Educators observed delayed asthma exacerbations several months after COVID-19 among children receiving asthma education for the first time. Characterization of this possible association will add to the body of knowledge regarding COVID-19 and asthma in children.

## 9 Biweekly delivery of food allergy-friendly grocery kits decreases food costs and increases mental health within 3 months

### Michael A. Golding^1,2^, Elissa M. Abrams^1,2,3^, Leslie E. Roos^1,2^, Moshe Ben-Shoshan^4^, Manvir Bhamra^1^, Justina Wiens^1^, Zoe Harbottle^1^, Jo-Anne St-Vincent^5^, Jennifer LP Protudjer^1,2,3^

#### ^1^The University of Manitoba, Winnipeg, MB; ^2^Children’s Hospital Research Institute of Manitoba, Winnipeg, MB; ^3^Karolinska Institutet, Stockholm, Sweden; ^4^McGill University, Montreal, QC; ^5^Children’s Allergy and Asthma Education Centre, Winnipeg, MB

##### **Correspondence:** Jennifer L. Protudjer

**Allergy, Asthma & Clinical Immunology** 2024, **20(Suppl 2)**:9

**Background:** Dietary avoidance is essential to prevent a potentially fatal allergic reaction. This near-constant need for dietary vigilance contributes to a social burden, including excess food costs and poorer mental health, for families who manage food allergy. Herein, we aimed to describe the financial and mental health impact of the first three months of a biweekly delivery of food allergy-friendly grocery kits to Winnipeg families managing milk allergy.

**Methods:** As part of an interventional study, we enrolled 10 Winnipeg families whose children aged ≤ 6 years with allergist-diagnosed milk allergy, and who had a net annual household income of less than $70,001. Families were recruited via social media and word-of-mouth. The intervention consisted of a bi-weekly, contactless home delivery (or elsewhere, by mutual agreement) of food allergy-friendly grocery kits, valued at ~ $75/kit. At baseline and midpoint (3 months after the start of the intervention), families completed a series of questionnaires on food costs and mental health.

**Results:** In total, the 10 participating families enrolled had an average monthly income of $3493.81. Surveys were completed exclusively by mothers. Children with milk allergy were age 3.0 ± 1.4 years, on average, and were ethnically diverse. In addition to milk, other food allergies were reported, most commonly to eggs, peanut and soy (n = 4 each). At baseline, monthly food costs averaged $736.36 ± $387.36, which decreased at midpoint to $673.75 ± 201.28, representing a non-statistically significant decrease of $62.61, or 8.5%. Regarding mental health from baseline to midpoint, generalized anxiety, depression and perceived stress did not change (all p ≥ 0.60). Mothers’ perceived life status, assessed using a visual analogue scale from 1–10, non-statistically significantly increased by 10.5% (baseline 6.82 ± 0.48; midpoint 8.0 ± 0.46).

**Conclusions:** Within three months, this unique intervention to support low-income families managing food allergy had reduced grocery costs, despite globally increasing food prices, and shows modest gains in perceived life status.

## 10 Parent feedback regarding virtual asthma, food allergy, and eczema education sessions during the Covid-19 pandemic

### Nancy Ross^1^, Shauna Filuk^1^, Jo-Anne St. Vincent^1^, Lenore Johnstone^1^, Caroline Doucette^1^, Elinor Simons^1,2^

#### ^1^The Children’s Allergy and Asthma Education Centre, Health Science Centre Winnipeg, Winnipeg, MB; ^2^Section of Allergy and Clinical Immunology and Children’s Hospital Research Institute of Manitoba, Department of Pediatrics and Child Health, University of Manitoba, Winnipeg, MB

##### **Correspondence:** Nancy Ross

**Allergy, Asthma & Clinical Immunology** 2024, **20(Suppl 2)**:10

**Background:** The Children’s Allergy & Asthma Education Centre (CAAEC) has a long history of providing in-person education to parents and children with asthma, food allergy and eczema. In April 2020, CAAEC in-person programing was halted due to the COVID-19 Pandemic and education sessions with asthma and allergy nurse educators were offered virtually. CAAEC sought to evaluate feedback from parents regarding this virtual education.

**Methods:** From June 2020 to June 2022, CAAEC received referrals from Emergency Departments, physicians’ offices, and self-referrals from the community to provide education to the families of children (0–17 years) with asthma, food allergy, and eczema. Parents were emailed a Microsoft Teams link for a 60-min session with a Certified Asthma Educator with experience in food allergy and eczema education. The nurse educators used PowerPoint slides and other visual aids to demonstrate correct inhaler, epinephrine auto-injector, and topical treatment techniques. Following the sessions, families were emailed electronic asthma, food allergy, and/or eczema resources and an anonymous SurveyMonkey® questionnaire link.

**Results:** CAAEC received 217 questionnaire responses for one or more of asthma (46.1%), food allergy (59.9%), and eczema (38.7%) education for children ages 0–6 years (83.9%), 7–11 years (12.9%), and 12–17 years (3.2%). Most participants reported that they had all their questions answered (97%), learned a great deal of new information (72%), found the information useful (87%) and clear (95%), and better understood how to manage (92%), and felt more confident in the management of (90%) their child’s asthma, food allergy, and eczema.

**Conclusions:** Virtual sessions with a qualified allergy nurse educator were a positive experience for parents, met their education needs, and improved their knowledge of and confidence in managing their child’s allergic condition. Virtual education continues to be offered as part of CAAEC programing. Further research evaluating its cost and accessibility would be useful.

**Acknowledgements**

We thank all the parents and families who completed education sessions and provided their anonymous feedback.

## 11 Evaluation of nurse-led eczema education sessions

### Jo-Anne St-Vincent^2^, Shauna Filuk^2^, Shannon Deane^1^, Elinor Simons^1^

#### ^1^Section of Allergy and Clinical Immunology and Children’s Hospital Research Institute of Manitoba, Department of Pediatrics and Child Health, University of Manitoba,, Winnipeg, MB; ^2^The Children’s’ Allergy and Asthma Education Centre, Winnipeg, MB

##### **Correspondence:** Jo-Anne St-VIncent

**Allergy, Asthma & Clinical Immunology** 2024, **20(Suppl 2)**:11

**Background:** Atopic dermatitis (AD) is associated with significant impairment of caregivers’ and children’s quality of life. Treatment for this inflammatory skin disease is not curative and long-term daily maintenance therapy is required to prevent chronic relapses. The CAAEC developed an education program to help caregivers gain knowledge and skills to manage AD. Our goal was to evaluate knowledge, behavior change, and caregiver satisfaction following an eczema education session.

**Methods:** The standardized Eczema Education Program was developed and directed towards children and teens with moderate-to-severe AD, and their caregivers. The program included a knowledge portion, direct demonstration of the application of emollients and prescribed medications, and specialized interventions such as wet wraps. Prior to April 2020, education was offered in person. Due to the COVID-19 Pandemic, most subsequent education sessions were provided virtually via Microsoft Teams video conferencing. A caregiver satisfaction questionnaire was emailed to families via Survey Monkey™ post education.

**Results:** Between June 2020 and June 2022, 23 participants responded to the questionnaire and reported:

- better ability to recognize early signs of eczema flare (87%),
- improved understanding of management strategies (82%)
- increased use of moisturizers (78%)
- fewer eczema flares (56%).
- that they would contact the educator in the future for questions and support (87%).

Information identified as “very useful” included: physiology of eczema, information about bathing, application of moisturizers, use of topical medicated creams, tips for managing itch and the availability of the educator for ongoing support.

**Conclusions:** AD education programs are rare and providing education can help to improve management of children’s AD. Information, treatment demonstration, and support provided to families can improve adherence to and efficacy of AD treatment. Virtual sessions are an effective way to increase knowledge and positively affect behavior change for eczema management. Educators play an ongoing role in supporting and educating families with AD.

## Food allergy/anaphylaxis

## 12 SMS messaging for patients on maintenance dosing for food allergen immunotherapy

### Alexandra V. Baaske^1^, Edmond S. Chan^1,2^, Raymond Mak^1,2^, Tiffany Wong^1,2^, Stephanie Erdle^1,2^, Rory L. Cunada^1,2^, Rishma Chooniedass^1,3^, Lianne Soller^1,2^

#### ^1^BC Children’s Hospital Research Institute, Vancouver, BC, ^2^Division of Allergy & Immunology, Department of Pediatrics, Faculty of Medicine, University of British Columbia, Vancouver, BC, ^3^School of Nursing, Faculty of Health and Social Development, University of British Columbia, Kelowna, BC

##### **Correspondence:** Alexandra V. Baaske

**Allergy, Asthma & Clinical Immunology** 2024, **20(Suppl 2)**:12

**Background:** BC Children’s Hospital Allergy Clinic provides longitudinal care to ensure patients are adherent and tolerating food allergy immunotherapy (FAIT). Scheduled follow-up visits occur during buildup and infrequently at maintenance, but between these appointments there is little communication between the patients and their healthcare team, relying on families to inform the clinic if there are issues. Increasing proactive communication with patients might reduce patient/caregiver anxiety and improve treatment adherence.

**Methods:** Patients who reached maintenance for FAIT between April 2021 and May 2022 were invited to receive bi-weekly text messages asking, “How are you?” via a healthcare communication platform called WelTel. Participant responses were categorized into “Okay” and “Not okay”. Healthcare providers monitored WelTel daily and responded to messages. Families were invited to complete surveys evaluating adherence, satisfaction, anxiety, and caregiver burden related to food allergy at the beginning of maintenance, and every 6 months thereafter.

**Results:** 136 families were approached for this study and 60 enrolled (44.1%). Of these, 57 parents and 3 adolescents received bi-weekly messages. The median age at start of FAIT was 6 years (IQR: 3.75, 11), and the most common foods treated were peanut (73.8%) and tree nuts (57.4%), with 53.3% on multiple foods. 46 families (76.7%) responded to at least one message. Two families withdrew from the study.

There were 271 total text conversations between families and healthcare providers, with 2.47 average messages per conversation. Most recognized responses were categorized as “Okay”. There was not enough data to analyze surveys quantitatively.

**Conclusions:** Automated text messaging might be a feasible way of increasing communication between patients and providers during maintenance of FAIT, where there is infrequent follow-up. This form of communication would not necessarily increase healthcare resource utilization. Future work will analyze effects on adherence and patient/caregiver outcomes such as anxiety, satisfaction, and caregiver burden.

## 13 Comparing relative allergen content and specific IgE binding in protein extracts from crushed peanut versus bamba

### Casey G. Cohen^1^, Wei Zhao^1^, Diana K. Toscano^1^, Danbing Ke^2^, Duncan Lejtenyi^2^, Liane Beaudette^2^, Christine McCusker^2^, Bruce D. Mazer^1,2^, Moshe Ben-Shoshan^2^

#### ^1^Research Institute of the McGill University Health Centre, Montreal, QC; ^2^Division of Allergy & Clinical Immunology, Department of Pediatrics, Montreal Children’s Hospital, Montreal, QC

##### **Correspondence:** Casey G. Cohen

**Allergy, Asthma & Clinical Immunology** 2024, **20(Suppl 2)**:13

**Background:** Peanut oral immunotherapy (OIT) could be achieved using crushed peanut or Bamba peanut snack (Osem, Israel). While the total peanut protein content in each food has been estimated, relative allergen content, extractability of proteins, and specific IgE binding have not been compared.

**Methods:** Raw peanut, roasted peanut, and Bamba were each processed into protein extracts and total protein concentrations were equilibrated. Samples were run on SDS-PAGE to analyze total protein content. Antibodies specific for allergens Ara h 1, Ara h 2, and Ara h 8 were used for detection in Western blot analyses and for relative quantification via ELISA. A pooled serum of 4 patients with high specific IgE for peanut (median sIgE: 474 kU/l, median age: 15 years old, 75% male) was used to quantify relative specific IgE binding via ELISA.

**Results:** Following the extraction of equal masses of food (Bamba or peanut) in equal volumes of buffer, Bamba samples yielded less extracted total protein when compared to raw (6.6-fold less) or roasted (3.3-fold less) samples. After adjusting total protein levels to equal concentrations, no significant differences in relative Ara h 1, Ara h 2, or Ara h 8 levels nor in specific IgE binding were found across all samples.

**Conclusions:** The results indicate similar allergen proportions in both Bamba and crushed peanut. However, less protein is extracted from equal masses of Bamba when compared to raw or roasted peanut, making Bamba a less potent and potentially safer substrate for peanut OIT.

## 15 Factors associated with tolerating a single-blind placebo-controlled food challenge in the pediatric population

### Luca Delli Colli^1^, Pasquale Mulé^1^, Casey Cohen^1^, Benjamin Lawson^1^, Sofianne Gabrielli^1^, Bruce Mazer^1^, Christine McCusker^1^, Danbing Ke^1^, Duncan Lejtenyi^1^, Liane Beaudette^1^, Edmond S. Chan^2^, Ingrid Baerg^2^, Julia Upton^3^, Eyal Grunebaum^3^, Philippe Bégin^4^, Ann E. Clarke^5^, Moshe Ben-Shoshan^1^

#### ^1^Division of Allergy and Clinical Immunology, Department of Pediatrics, Montreal Children’s Hospital, McGill University Health Centre, Montreal, QC; ^2^Division of Allergy and Immunology, Department of Pediatrics, BC Children’s Hospital, University of British Columbia, Vancouver, BC; ^3^Division of Immunology and Allergy, Department of Pediatrics, The Hospital of Sick Children, University of Toronto, Toronto, ON, ^4^Division of Allergy and Clinical Immunology, Department of Pediatrics, Centre Hospitalier Universitaire Sainte-Justine, Université de Montréal, Montreal, QC; ^5^Division of Rheumatology, Department of Medicine, University of Calgary, Calgary, AB

##### **Correspondence:** Luca Delli Colli

**Allergy, Asthma & Clinical Immunology** 2024, **20(Suppl 2)**:15

**Background:** The factors associated with tolerance of a food challenge remain unclear. We aimed to assess factors associated with tolerating a single-blind placebo-controlled food challenge (SBPCFC) in the pediatric population.

**Methods:** Patients were recruited from the Montreal Children’s Hospital, British Columbia Children’s Hospital, the Hospital for Sick Children, and Hôpital Sainte-Justine. Children above 6 years old with physician-diagnosed food allergy to milk, peanut, hazelnut, and/or egg, and a convincing history of reaction to the food were deemed eligible for recruitment [1]. Prior to beginning oral immunotherapy (OIT), all patients underwent a SBPCFC, as oral food challenges are the gold standard for diagnosis of food allergy. Factors associated with tolerating SBPCFC were assessed using a multivariate regression.

**Results:** Over 9 years, a total of 168 children were recruited to participate in OIT. The median age was 10.5 [Interquartile range (IQR) 8.0, 14.0] and 54.8% were male. SBPCFC to milk, egg, peanut, and hazelnut were tolerated in 7 (6.80%), 1 (5.00%), 2 (6.25%), and 7 (53.8%) patients, respectively. Previous epinephrine use was reported in 38.3% of patients challenged to milk, 21.7% of patients challenged to egg, 34.4% of patients challenged to peanut, and 7.69% challenged to hazelnut.

Tolerating SBPCFC was more likely in patients challenged to hazelnut [adjusted Odds Ratio (aOR) 1.41 (95% CI, 1.22, 1.64)], and in those with lower baseline sIgE levels [aOR 0.998 (95% CI, 0.997, 0.999)] while adjusting for sex, age, and baseline skin prick test measurement.

**Conclusions:** Compared to other food allergens, children with physician-diagnosed hazelnut allergy are more likely to tolerate the culprit allergen and hence, a food challenge is crucial prior to hazelnut OIT initiation.

## References

Ben-Shoshan M, Harrington DW, Soller L, Fragapane J, Joseph L, St Pierre Y, et al. A population-based study on peanut, tree nut, fish, shellfish, and sesame allergy prevalence in Canada. J Allergy Clin Immunol. 2010;125(6):1327-35

## 17 Modified sesame desensitization protocol in real-world clinical practice

### Sofianne Gabrielli^1^, Pasquale Mulé^1^, Dima Elgendy^1^, Karen Sigman^1^, Christine McCusker^1^, Sarife Saker^1^, Xun Zhang^2^, Moshe Ben-Shoshan^1^

#### ^1^Division of Pediatric Allergy and Clinical Immunology, Department of Pediatrics, McGill University Health Centre, Montreal, QC; ^2^Centre for Outcomes Research and Evaluation, Research Institute of McGill University Health Centre, Montreal, QC

##### **Correspondence:** Sofianne Gabrielli

**Allergy, Asthma & Clinical Immunology** 2024, **20(Suppl 2)**:17

**Background:** Sesame and sesame-containing foods have become increasingly prevalent in Western diet, meaning that patients allergic to sesame are at risk for severe, life-threatening reactions. We aimed to assess the efficacy and safety of a modified sesame desensitization protocol in children in real-world clinical practice.

**Methods:** Children with a history of sesame allergy and a positive skin prick test to sesame were recruited at the Montreal Children’s Hospital and the Children’s Clinic located in Montreal. After obtaining consent, an initial dose of sesame protein (3–25 mg) was introduced in the form of either tahini muffin or sesame seeds. Patients continued the same dose for 2–5 weeks at home, filled out a symptom diary, and returned to the clinic for updosing until maintenance was reached (2 teaspoons of hummus or 2 mL of tahini). A non-parametric test (Wilcoxon rank sum test) was used to compare the numbers of allergic reactions between tahini muffin and sesame seed protocols.

**Results:** Between January 2021 and May 2022, 31 children (58.1% male; median age 2.5 years) were recruited. The majority of patients (77.4%) has eczema, 32.3% had asthma, and 81.3% had other food allergies (mainly peanut (61.5%), cashew (42.3%), and hazelnut (30.8%)). Oral desensitization was performed using one of two strategies according to the allergist: initial doses were either tahini muffin (69.2%) or sesame seeds (41.9%). To date, 7 patients (22.6%), after an average of 7.4 visits, reached the maintenance dose. Among the 8,515 total intake doses of sesame, 13 cases of non-anaphylactic allergic reactions and 1 case of anaphylaxis occurred. Over half (57.1%) of the reactions occurred at clinic during first visit or updosing. There were significantly more non-anaphylactic allergic reactions to sesame seeds than to tahini muffin (p-value = 0.016).

**Conclusions:** Modified sesame desensitization protocols can be safely used in children with sesame allergy.

## 19 Safety and efficacy of early oral immunotherapy for peanut allergy in a primary care setting

### Victoria Landry^1^, Christopher Vaillancourt^1,2,3^, Kavish Chandra^1,2,3^, Rachel Lewis^4^, William Lewis^4^, Jacqueline Fraser^2,3^, Andrew Flewelling^3^, Paul Atkinson^1,2,3^

#### ^1^Dalhousie University, Saint John, NB; ^2^Saint John Regional Hospital Department of Emergency Medicine, Saint John, NB; ^3^Horizon Health Network, Saint John, NB, ^4^Dalhousie University, Halifax, NS

##### **Correspondence:** Victoria Landry

**Allergy, Asthma & Clinical Immunology** 2024, **20(Suppl 2)**:19

**Background:** Peanut allergy is a common food allergy that can cause life threatening anaphylaxis. Early oral immunotherapy for peanut allergy (P-EOIT) has been shown to be effective and safe in research and specialty clinic settings. New Brunswick has limited access to specialty allergy care, and provision of P-EOIT in primary care would make it available to more patients. We sought to assess the safety and efficacy of successful P-EOIT completion in a primary care setting, along with rates of peanut-related allergic reactions leading to emergency department (ED) visits and use of epinephrine.

**Methods:** This study included all patients starting P-EOIT under 36 months old at a primary care allergy clinic in New Brunswick, Canada from 2016 to 2020. Patients had a history of allergic reaction to peanut with a positive skin prick test or positive peanut specific IgE level (ps-IgE), or ps-IgE ≥ 5 kU/L with no history of peanut allergy. Patients had biweekly clinic visits with graded increase in peanut protein up to a maintenance dose of 300 mg peanut protein daily. A blinded retrospective chart review was conducted along with phone interviews regarding ED visits and epinephrine use. This study was approved by our Regional Research Ethics Board.

**Results:** Of 69 consented patients, 67 (97.1%) were followed up successfully. All consented patients reached maintenance dose, over a median of 29 weeks, and 66 patients (95.7%) were still regularly consuming peanut protein after one year of maintenance. One patient had a peanut ingestion-related ED visit requiring epinephrine during the escalation phase of peanut protein dose (1.5%; 95% CI 0.0 to 8.0%). During the first year of maintenance phase, no patients had peanut ingestion-related ED visits or required epinephrine.

**Conclusions:** P-EOIT in a primary care setting appears to be safe and effective with the majority of patients reaching the maintenance phase.

## 20 The effectiveness of oral immunotherapy on food allergy related quality of life

### Benjamin J. Lawson^1^, Pasquale Mulé^1^, Vera Laboccetta^1^, Luca Delli Colli^1^, Greg Shand^1^, Christine McCusker^1,2^, Karen Sigman^1,2^, Sarife Saker^1,2^, Xun Zhang^1,2^, Moshe Ben-Shoshan^1,2^

#### ^1^Division of Allergy and Clinical Immunology, Department of Pediatrics, McGill University Health Centre, Montreal, QC; ^2^Centre for Outcomes Research and Evaluation, Research Institute of McGill University Health Centre, Montreal, QC, Canada, Montreal, QC

##### **Correspondence:** Benjamin J. Lawson

**Allergy, Asthma & Clinical Immunology** 2024, **20(Suppl 2)**:20

**Background:** Food allergies have become increasingly common in North America, and place many patients at risk of severe, life-threatening allergic reactions. A lower health-related quality of life may be experienced by families with food-allergic children as a result of anxiety related to the possibility of accidental exposure and psychosocial consequences. We seek to assess the effect of successful Oral Immunotherapy (OIT) on parent-reported quality of life given a growing body of evidence that shows it can greatly reduce the risks associated with food allergies.

**Methods:** Children with a convincing history of allergy diagnosed via a positive skin prick test to either sesame, peanut, or walnut were recruited at the Montreal Children’s Hospital and The Children’s Clinic located in Montreal. Parents of participants were assessed using a Food Allergy Quality of Life Questionnaire-Parent Form (FAQLQ-PF) at baseline prior to desensitization, and again after participants had reached maintenance (Either 2 teaspoons of hummus, ¼ teaspoons of peanut butter, or 1/8 walnut, dependent on allergen). A non-parametric test was conducted to detect differences in the scores at baseline and at follow-up.

**Results:** 14 Children (57% female, median age = 1.8 years) were included in the study. Participants had undergone desensitization for peanut (79%), sesame (14%) and walnut (7%). No significant difference between total FAQLQ-PF scores at baseline and at follow up were found (p = 0.391). Similarly, no significant differences were found in the scores of the Food Anxiety (p = 0.906), Emotional Impact (p = 0.149), and Social/Dietary Restriction (p = 0.972) subscales. A significant difference was found between the baseline and follow-up scores of the subscale assessing parental concern regarding accidental exposure (p = 0.037), but not in the subscale assessing the child’s level of concern regarding accidental exposure (p = 0.447).

**Conclusions:** Our findings suggest that parental concern is significantly affected by pediatric OIT, but global Health-Related Quality of Life (HRQL) is not.

## 22 Long-term adherence and risk of allergic reactions in children who attained milk oral immunotherapy maintenance

### Pasquale Mulé^1^, Bruce Mazer^1^, Danbing Ke^1^, Lydia Zhang^1^, Duncan Lejtenyi^1^, Chiara Lucchesi^1^, Liane Beaudette^1^, Luca Delli Colli^1^, Julia Upton^2^, Eyal Grunebaum^2^, Ciriaco Piccirillo^3^, Edmond S. Chan^4^, Ann Clarke^5^, Xun Zhang^1^, Sofianne Gabrielli^1^, Christine McCusker^1^, Moshe Ben-Shoshan^1^

#### ^1^Division of Allergy and Clinical Immunology, Department of Pediatrics, Montreal Children’s Hospital, McGill University Health Centre, Montreal, QC; ^2^Division of Immunology and Allergy, Department of Pediatrics, The Hospital for Sick Children, University of Toronto, Toronto, ON; ^3^Department of Microbiology and Immunology, McGill University Health Center, Montreal, QC; ^4^Division of Allergy and Immunology, Department of Pediatrics, BC Children’s Hospital, University of British Columbia, Vancouver, BC; ^5^Division of Rheumatology, Department of Microbiology and Immunology, Cumming School of Medicine, University of Calgary, Calgary, AB

##### **Correspondence:** Pasquale Mulé

**Allergy, Asthma & Clinical Immunology** 2024, **20(Suppl 2)**:22

**Background:** Data on long-term adherence and efficacy of oral immunotherapy (OIT) is sparse. We aimed to assess long-term adherence and the risk of allergic reaction after attaining OIT maintenance.

**Methods:** Thirty-six children who reached their OIT maintenance dose of 200 ml milk were followed at the Montreal Children’s Hospital. Patients were queried annually on milk consumption and allergic reactions. Survival analysis was performed to evaluate the association between the risk of reaction and adherence to OIT.

**Results:** Patient ages ranged from 8 to 21 years old (median 15, 50% male). Median follow-up time after beginning maintenance was 2.5 years (range 0.4—5.4 years). Eighteen patients (50%) adhered to OIT protocol (≥ 200 ml milk, ≥ 3 times per week), 3 patients (8%) partially adhered (< 200 ml milk or baked goods, ≥ 3 times per week), 6 patients (17%) did not adhere (dairy products 1–2 times per week), and 9 patients (25%) stopped ingesting dairy products.

Reactions occurred in 13 patients (36%). 3/13 (23%) reactions occurred following maintenance dose ingestion and 10/13 (77%) reactions occurred following dietary exposure (cheese and baked goods). Exercise was the only cofactor (38% of reactions). Reactions occurred in 2/18 (11%) adherent patients, in which one was anaphylaxis. Reactions occurred in 11/18 (61%) patients who deviated from protocol, 8 of which were anaphylaxis. No association was observed between risk of reaction and sex (hazard ratio (HR) = 1.05, 95% CI [0.35–3.16]), or age (HR = 1.06, 95% CI [0.90–1.24]). Adherent patients had lower risk of reaction (HR = 0.41, 95% CI [0.09–1.88]), although this effect was not statistically significant.

**Conclusions:** Half of our cohort adhered to OIT protocol, and reactions occurred in approximately a third of patients. While adherent patients had a lower risk of reaction, the small sample size precluded definitive conclusions.

## 23 In vitro sublingual protein penetration rates of peanut sublingual immunotherapy preparations

### Brock A. Williams, Yigong Guo, Lianne Soller, Edmond S. Chan, Anubhav Pratap-Singh

#### The University of British Columbia, Vancouver, BC

##### **Correspondence:** Brock A. Williams

**Allergy, Asthma & Clinical Immunology** 2024, **20(Suppl 2)**:23

**Background:** Sublingual immunotherapy (SLIT) is a safe therapy which can be used in the treatment of peanut allergy. Studies demonstrating peanut SLIT efficacy have relied on glycerinated food extracts for the administration of food allergens. These extracts are costly, not licensed for SLIT, and largely unavailable for patient use at home, thus limiting the accessibility of this treatment. Objective: To determine the in vitro sublingual protein penetration rates of commercially available peanut powder, in comparison with glycerinated peanut extract (the current standard in research).

**Methods:** Defatted peanut powder (~ 6 g protein per 15 g serving) and glycerinated peanut extract (1:20 w/v fully dissolved in 0.2% phenol and 50% glycerinated saline) were tested for in vitro sublingual protein penetration rates using human buccal carcinoma cell lines (TR 146). To determine protein penetration rates, samples from the basolateral chamber of the TR 146 cell line were obtained at 0.5, 1, 2, and 4 h after inoculation with 37 ^◦^C incubation. The permeated amount of protein from extract was analyzed by Bradford protein assay.

**Results:** Glycerinated peanut extract showed higher protein penetration rates at 0.5 h (36.7 ± 1.2% vs. 30.3 ± 1.5%), 1 h (82.2 ± 2.0% vs. 72.1 ± 1.5%) and 2 h (89.8 ± 1.5% vs. 80.0 ± 2.0%) of incubation compared with peanut powder (p < 0.05), but not after 4 h (95.5 ± 1.5% vs. 96.1 ± 1.5%, respectively) (p > 0.05).

**Conclusions:** Glycerinated peanut extracts had higher sublingual penetration rates from 0.5–2 h of incubation than peanut powder. Further work using in vitro and in vivo methods to establish the potential use of widely accessible peanut powder as a therapeutic in peanut SLIT is warranted.

## 25 Using the Canadian egg ladder in canadian children with food protein-induced enterocolitis syndrome (FPIES) to egg

### Linlei Ye^1^, Victoria E. Cook^2^, Stephanie C. Erdle^2^

#### ^1^Department of Pediatrics, University of Alberta, Edmonton, AB, ^2^Division of Allergy and Immunology, University of British Columbia, Vancouver, BC

##### **Correspondence:** Linlei Ye

**Allergy, Asthma & Clinical Immunology** 2024, **20(Suppl 2)**:25

**Background:** Current management of food protein-induced enterocolitis syndrome (FPIES) involves strict avoidance of the offending food for 12–18 months, followed by oral food challenge under physician supervision. Prolonged avoidance may increase the risk of IgE-mediated allergy, particularly in atopic patients. Food ladders have shown success in promoting accelerated tolerance in patients with IgE-mediated allergy. Our case series evaluated the safety of use of the Canadian Egg Ladder in patients with FPIES to egg.

**Methods:** From May 2020 to November 2021, patients with mild FPIES to egg, defined as no history of lethargy or intravenous fluid administration, were started on the Canadian Egg Ladder. Patients were followed every 3—6 months, at which time information was collected regarding progression up the ladder, symptoms while on treatment, and interventions required. Treating allergists completed a survey to capture baseline demographic characteristics and prior tolerance to egg. Descriptive statistics were analyzed using MS Excel.

**Results:** 21 patients with mild FPIES were started on the Canadian Egg Ladder. Median age at initiation of the ladder was 10 months (IQR, 9–11). Seventeen (80.9%) patients successfully completed the ladder, tolerating a serving size amount of cooked egg, with a median duration of 7 months (IQR, 4–9). Four (19.0%) patients experienced symptoms while undergoing treatment including vomiting (9.5%), pallor (9.5%), belching (4.8%), and irritability (4.8%). There were no reports of lethargy. No patients required health care presentation or intravenous fluid administration. No patients discontinued the ladder.

**Conclusions:** The Canadian Egg Ladder can safely guide the gradual advancement of egg-containing foods in patients with mild FPIES to egg, without needing prolonged avoidance and resource intensive oral food challenges. Promoting consistent consumption of egg may reduce the risk of developing IgE-mediated egg allergy. Larger scale prospective studies are planned to confirm these promising results.

## Immunology

## 26 Cross-sectional study of patients with secondary immunodeficiency: Ontario Immunoglobulin Treatment (ONIT) case registry data

### Armin Abadeh^1^, Stephen Betschel^1^, Susan Waserman^2^, Donald W. Cameron^3^, Juthaporn Cowan^3^

#### ^1^University of Toronto, Department of Medicine, Toronto, ON; ^2^McMaster University, Department of Medicine, Hamilton, ON, 3. University of Ottawa, Department of Medicine, Ottawa, ON

##### **Correspondence:** Armin Abadeh

**Allergy, Asthma & Clinical Immunology** 2024, **20(Suppl 2)**:26

**Background:** Despite the increasing number of cases of secondary immunodeficiency and immunoglobulin utilization, there is a paucity of data in the literature on clinical outcomes and patient-reported health-related QoL measures in this population. This is the first report from the Ontario Immunoglobulin Treatment (ONIT) registry, a multicentre program based at Ottawa, Hamilton and Toronto, on patients with secondary immunodeficiency.

**Methods:** Cross-sectional study of patients with secondary immunodeficiency enrolled in the ONIT Case Registry from June 2020 to March 2022 was completed. Demographics, comorbidities, indication for immunoglobulin treatment, clinical data infections, and patient-reported quality of life parameters were collected and analyzed.

**Results:** A total of 108 patients (45 male; 63 female; average age of 66) with secondary immunodeficiency as the main indication for IG treatment were identified. Of these patients, 93 were on SCIG (of whom 19 were previously on IVIG) and 15 were on IVIG. The most common indication was CLL (N = 33), followed by lymphoma (N = 20) and transplant (N = 11). 92% of patients were followed by one or more subspecialists, with the most common being hematology/oncology (62%). SCIG average dosage was 0.57 ± 0.30 g/kg/4 weeks and IVIG average dosage was 0.49 ± 0.15 g/kg/4 weeks. IG treatment reduced average annual number of infections by 83% (4.3 vs. 0.7) and the number of emergency department visits by 85% (1.3 vs 0.2). 91.7% of the patients who switched from IVIG to SCIG reported their health as same or better compared to before the switch. Overall, 80.5% of patients reported their health as better compared to before they started immunoglobulin treatment.

**Conclusions:** To our knowledge, this is the largest cross-sectional descriptive study reported on patients with secondary immunodeficiency receiving IG treatment in Canada. IG treatment has improved clinical and quality of life reported outcomes. The data reported here can be invaluable in improving care coordination and management of these complex patients.

## 27 Validating the intra-assay and inter-assay coefficient of variation for the MILLIPLEX® SARS-CoV-2 Immunoglobulin-A,G,M magnetic bead based immunoassays

### Alyssa G. Burrows^1,2^, Jenny Thiele^2,3^, Sophia Linton^1,2^, Prameet M. Sheth^3,4,5^, Stephen Vanner^1,4^, Anne K. Ellis^1,2,3^

#### ^1^Department of Medicine, Queen’s University, Kingston, ON; ^2^Allergy Research Unit, Kingston, ON; ^3^Department of Biomedical and Molecular Sciences, Queen’s University,, Kingston, ON; ^4^Gastrointestinal Disease Research Unit (GIDRU), Faculty of Health Science, Queen’s University, Kingston, ON, ^5^Pathology & Molecular Medicine, Queen’s University,, Kingston, ON

##### **Correspondence:** Alyssa G. Burrows

**Allergy, Asthma & Clinical Immunology** 2024, **20(Suppl 2)**:27

**Background:** Globally, severe-acute-coronavirus disease-2(SARS-CoV-2) has caused over 500 million infections and over 6 million deaths, respectively [1]. Monitoring SARS-CoV-2 seroprevalence is crucial to determining population immunity.

**Methods:** Serum samples were collected prospectively from Queen’s University Faculty of Health Sciences(FHS) students and recovered community members to determine asymptomatic carriage and seroprevalence to SARS-CoV-2. A blank, recovered, vaccinated and negative control samples were selected and run in duplicate with the SARS-CoV-2 immune-globulin(Ig)-A,M,G MILLIPLEX®(MilliporeSigma) Luminex®XMap® magnetic bead-based immunoassays to measure antibodies to four SARS-CoV-2 proteins(spike-1(S1), spike-2(S2), nucleocapsid(N), receptor-binding-domain(RBD)) and four control beads(Control Bead(CB)-1, CB2, CB3, Negative Control Bead). With the goal of validating the intra- and inter-assay %CV across a larger sample size of assays and comparing them to the manufacturer’s provided intra- and inter-assay %CV of < 15% and < 20%, respectively. The assays were read with Bio-Plex Manager™ Software6.2 and calculations were done with Excel(2016).

**Results:** Between May 2020-June 2021, 1,229 and 16 serum samples were collected from 457 FHS participants and 11 recovered community participants, respectively. The mean intra-assay %CV for the SARS-CoV-2 proteins ranged from 5.50–7.14, 4.49–5.87, and 4.67–6.67 for IgA(n = 14), IgM(n = 14), IgG(n = 15), respectively. The mean intra-assay %CV for the four control beads ranged from 2.83–9.67, 3.24–5.73, and 1.27–10.23 for IgA(n = 14), IgM(n = 14), IgG(n = 15), respectively. The mean inter-assay %CV for the SARS-CoV-2 proteins ranged from 8.69–11.02, 9.10–11.20, 7.52–11.71 for IgA(n = 14), IgM(n = 14), IgG(n = 15), respectively after the removal of outliers (S1 for negative control sample for IgA and IgG and RBD for blank well for IgG).

**Conclusions:** The mean intra-assay %CV for all targets were < 10% except for IgG for CB3. The mean inter-assay %CV were < 12% after the removal of the aforementioned outliers. The intra- and inter-assay %CV were lower than the manufacturer’s reported < 15% and < 20%.

COVID-19 Map [Internet]. Johns Hopkins Coronavirus Resource Center. [cited 2022 May 5]. Available from: https://coronavirus.jhu.edu/map.html.

## 28 Genetic testing for inborn errors of immunity: comparing a primary immunodeficiency panel with a comprehensive immune and cytopenia panel

### Christine Davies^1^, Allison Sluyters^1^, Zoe Powis^1^, Kimberly Gall^1^, Julie Hathaway^1^, Alicia Scocchia^1^, Elina Hirvonen^2^, Päivi Kokkonen^2^, Inka Saarinen^2^, Matias Rantanen^2^, Pertteli Salmenperä^2^, Massimiliano Gentile^2^, Jennifer Schleit^1^, Lotta Koskinen^2^, Jussi Paananen^2^, Samuel Myllykangas^2^, Juha Koskenvuo^2^

#### ^1^Blueprint Genetics, Seattle, WA, USA, 2. Blueprint Genetics, Espoo, Finland

##### **Correspondence:** Christine Davies

**Allergy, Asthma & Clinical Immunology** 2024, **20(Suppl 2)**:28

**Background:** Identifying the genetic etiology of inborn errors of immunity (IEI) significantly impacts management but can be challenging if the primary immunodeficiency (PID) overlaps with hematological disorders like cytopenia and bone marrow failure. A Comprehensive Immune & Cytopenia (CIC) next-generation sequencing panel was developed to address this need. We compared the results of this panel to those from a PID panel during the same period.

**Methods:** A retrospective review of deidentified results from 1,243 consecutive patients tested with the CIC (642 genes) or the PID (298 genes) panel was performed. Test results, patient age, clinical information and panel used were extracted. The PID and the CIC panels contain 7 and 313 unique genes respectively and share 329 genes in common. Target regions included all coding exons (unless otherwise indicated), 20 base pairs at intron–exon boundaries, and select noncoding variants. Copy number variants (CNVs) were analyzed bioinformatically with a proprietary pipeline to detect exon-level CNVs. Variant interpretation was performed using a modification of the ACMG guidelines.

**Results:** The *BTK* (9%) and *STAT3* (6%) genes made up most diagnoses for the cohort tested with the PID panel. For the CIC panel, the gene responsible for the most diagnoses was *XIAP* (9%). CNVs were responsible for the diagnosis in 15% of patients while noncoding variants were responsible for the diagnosis in 7% of patients. Variants in difficult-to-sequence genes accounted for 10% of the diagnoses.

**Conclusions:** We demonstrate the clinical utility of genetic testing for IEI and its hematologic phenocopies. While both panels had a similar yield, the most frequent diagnostic genes are panel specific. CNVs, noncoding variants, and difficult-to-sequence genes are important contributors to the diagnostic potential highlighting the value of panels with high-resolution CNV capabilities, methods to resolve challenging regions, and the inclusion of clinically relevant noncoding variants for this patient population.

## 29 Dupilumab reduces biomarkers of type 2 inflammation in adult and adolescent patients with eosinophilic esophagitis in parts a and c of a three-part, phase 3 lLIBERTY EoE TREET study

### Marc Rothenberg^2^, Ikuo Hirano^3^, Evan S. Dellon^4^, John Leung^5^, Jason Lee^1^, Angela Khodzayev^6^, Juby A. Jacob-Nara^7^, Danen M. Cunoosamy^8^, Jennifer D. Hamilton^6^

#### ^1^Clinical Immunology and Allergy and Internal Medicine, Toronto Allergy and Asthma Clinic, Toronto, ON; ^2^Cincinnati Children’s Hospital Medical Center and University of Cincinnati College of Medicine, Cincinnati, OH, USA; ^3^Northwestern University Feinberg School of Medicine, Chicago, IL, USA, ^4^University of North Carolina School of Medicine, Chapel Hill, NC, USA; ^5^Tufts Medical Center, Boston, MA, USA; ^6^Regeneron Pharmaceuticals, Inc, Tarrytown, NY, USA; ^7^Sanofi, Bridgewater, NJ, USA, ^8^Sanofi, Cambridge, MA, USA

##### **Correspondence:** Jason Lee

**Allergy, Asthma & Clinical Immunology** 2024, **20(Suppl 2)**:29

**Background:** Dupilumab, a fully human monoclonal antibody, blocks the shared receptor component for interleukin (IL)-4 and IL-13. Part A of 3-part, phase 3 LIBERTY-EoE-TREET (NCT03633617) demonstrated the efficacy and safety of weekly dupilumab 300 mg vs placebo in adolescent and adult patients with eosinophilic esophagitis (EoE) for 24 weeks. For patients completing Part A, Part C was a 28-week extended active treatment period to evaluate efficacy and safety of weekly dupilumab 300 mg for 52 weeks. This analysis assessed dupilumab effect on biomarkers of type 2 inflammation in Part C.

**Methods:** Of 81 patients (42 dupilumab; 39 placebo) enrolled in Part A, 77 continued to dupilumab in Part C (40 dupilumab [dupilumab/dupilumab]; 37 placebo [placebo/dupilumab]). Median changes from Part A baseline (∆BL) in serum thymus and activation-regulated chemokine (TARC), plasma eotaxin-3, and serum total IgE were assessed.

**Results:** Part A BL biomarker levels were similar across treatment groups. In Part A, at Week 24, median ∆BL(Q1,Q3) for dupilumab vs placebo in TARC was -115.5 pg/mL(− 204.0, − 60.0) vs -35.0 pg/mL(− 67.0,32.0) (Part A BL values[Q1,Q3]: 322.0 pg/mL[232.0,430.0] vs 293.0 pg/mL[226.0,418.0]); eotaxin-3 was -88.6 pg/mL(− 212.0,-47.0) vs − 9.0 pg/mL(− 148.0,53.0) (Part A BL values[Q1,Q3]: 217.5 pg/mL [139.0,330.0] vs 217.0 pg/mL[163.0,448.0]); and total IgE was -45.7kU/L(− 198.0, − 23.7) vs − 8.6kU/L(− 72.0,4.7) (Part A BL values[Q1,Q3]: 110.0kU/L[51.1,463.0] vs 100.0kU/L[46.7,294.0]) (all nominal *P* < 0.0001). In Part C, at Week 52, median ∆BL(Q1,Q3) for dupilumab/dupilumab and placebo/dupilumab in TARC was -98.0 pg/mL(-182.0,-37.0) and − 122.0 pg/mL(− 194.0, − 28.0); eotaxin-3 was − 118.0 pg/mL(− 225.3, − 63.8) and − 160.9 pg/mL(− 367.0, − 104.6); and total IgE was − 62.9kU/L(− 410.0,-35.4) and − 57.6kU/L(− 178.8, − 28.7). Dupilumab demonstrated an acceptable safety profile in Part C; TEAEs occurring ≥ 10% in dupilumab/dupilumab and placebo/dupilumab were injection-site reactions (10.0% and 21.6%) and injection-site erythema (10.0% and 13.5%).

**Conclusions:** Dupilumab suppressed TARC, eotaxin-3, and total IgE in EoE patients over 52 weeks, consistent with prior assessment in EoE and other type 2 inflammatory diseases. Placebo/dupilumab patients in Part C showed similar treatment effects to dupilumab patients in Part A.

## 30 Long term natural history of Good’s Syndrome

### Vanessa Polito, Aunonna Kabir, Christos Tsoukas

#### McGill University, Montreal, QC

##### **Correspondence:** Vanessa Polito

**Allergy, Asthma & Clinical Immunology** 2024, **20(Suppl 2)**:30

**Background:** Good’s syndrome is a rare adult-onset combined immune deficiency, characterized by thymoma and hypogammaglobulinemia. Patients present late in disease, following recurrent sinopulmonary infections, opportunistic infections, cytopenias, absent B cells and T-cell abnormalities. Documentation of the long term clinical and laboratory evolution is lacking due to the rarity of the disease and the relative lack of cohort studies.

**Methods:** We report a longitudinal, single site, case series of seven patients from 1980 to present. Demographics, clinical presentation including age at key events including: hypogammaglobulinemia, immunoglobulin replacement, and death were collected along with the type and frequency of infections, and thymoma pathology. Clinical and laboratory evolution over time, including quantitative immunoglobulins, complete blood count, B and T cell numbers and function were documented.

**Results:** Mean age at thymoma diagnosis was 51, of hypogammaglobulinemia was 50, and of immunoglobulin replacement was 58.5. In 5/7 patients, thymoma pathology was WHO type AB. All patients had absent B cells, and all but one patient were severely hypogammaglobulinemic. All but one patient developed recurrent sinopulmonary infections prior to the thymoma. All demonstrated in vitro and in vivo T cell dysfunction, on either lymphocyte proliferation testing or delayed-type hypersensitivity skin testing. Clinical history was notable for opportunistic and chronic bacterial infections. Cytopenias appeared to emerge with advanced disease in 4/7 patients. Two patients are deceased: one patient from progressive multifocal encephalopathy due to polyoma virus at age 69, another with systemic cytomegalovirus and subsequent septic shock from pseudomonas pneumonia at age 71.

**Conclusions:** During the last 4 decades, we systematically documented the natural history of seven patients with Good’s syndrome. Clinical and laboratory evolution of these patients provides crucial longitudinal information about the disease progression and will support efforts to better characterize this rare disease.

## 32 Stimulation of human mucosal B cells with germinal center cytokines results in the generation of Il-10 + regulatory plasmablasts

### Nicholas Vonniessen, Eisha Ahmed, Jana Abi Rafeh, Bruce Mazer

#### Meakins-Christie Laboratories, McGill University, Montreal, QC

##### **Correspondence:** Nicholas Vonniessen

**Allergy, Asthma & Clinical Immunology** 2024, **20(Suppl 2)**:32

**Background:** The ability of B cells to regulate immunity, beyond antibody production, resulted in the classification of a novel subset of B-cells, called regulatory B-cells (Breg). The identification of this subset was initially based on their ability to attenuate inflammation through the production of IL-10. Considering their critical role in promoting peripheral tolerance and suppressing the development of type II inflammatory responses that drive allergic disease, we sought to evaluate whether Th2 GC conditions were capable of inducing the expansion of IL-10 + Breg using human mucosal B cells.

**Methods:** Human tonsillar B cells were cultured for 2 to 5 days at a concentration of 2 × 10^6^ cells/ml in 12-well plates. Cells were stimulated with or without anti-CD40 (1ug/mL, purified from the G28.5 cell line), IL-4 (100U/mL), IL-21 (50 ng/mL) or CpG ODN 2006 (5ug/mL). For intracellular cytokine detection by flow cytometry, 50 ng/ml PMA, 1ug/ml ionomycin and 2uM GolgiStop were added for the last 5 h of culture. IL-10 secretion was assessed by ELISA and ELISpot.

**Results:** Compared to CpG + anti-CD40, a well-cited method for the induction of human regulatory B cells, stimulation with anti-CD40 + IL-4 + IL-21 resulted in a robust induction of CD19 + IL-10 + Breg. Class-switched plasmablasts defined the primary Breg subset within the CD19 + IL-10 + CD27 + IgD- population in response to both CpG + anti-CD40 and anti-CD40 + IL-4 + IL-21 on Day 2; however, this population persisted until Day 5 only in response to stimulation with anti-CD40 + IL-4 + IL-21.

**Conclusions:** Results identified anti-CD40 + IL-4 + IL-21 as a novel highly effective condition for the induction of human regulatory B cells and suggests a role for these cells in the regulation of Th2 GC responses. The induction and maintenance of regulatory plasmablasts in response to GC cytokines raises questions regarding the role of these cells in the suppression of IgE-mediated inflammation and whether a deficiency in this response may contribute to the pathophysiology that characterizes allergic disease.

## Other allergy/immunology

## 33 Vaccine confidence amongst those living with allergy during the Covid pandemic (ACCORD): a scoping review

### Ayel Luis R. Batac^1,2^, Kaitlyn A. Merrill^1,2^, Nicole Askin^3^, Michael A. Golding^1,2^, Elissa M. Abrams^1,2,4^, Philippe Bégin^5,6^, Moshe Ben-Shoshan^7,8,9^, Erika Ladouceur^10^, Leslie Roos^11^, Vladan Protudjer^12^, Jennifer LP Protudjer^1,2,13^

#### ^1^Department of Pediatrics and Child Health, Max Rady College of Medicine, Rady Faculty of Health Sciences, University of Manitoba, Winnipeg, MB; ^2^Children’s Hospital Research Institute of Manitoba, Winnipeg, MB; ^3^WRHA Virtual Library, Winnipeg, MB; ^4^Division of Allergy and Immunology, Department of Pediatrics, Faculty of Medicine, University of British Columbia, Vancouver, BC, ^5^Division of Allergy, Department of Pediatrics, Centre hospitalier universitaire Sainte-Justine, Montreal, QC, ^6^Division of Allergy, Department of Medicine, Centre hospitalier de l’Université de Montréal, Montreal, QC; ^7^Division of Pediatric Allergy, Clinical Immunology and Dermatology, Department of Pediatrics, Montreal Children’s Hospital, Montreal, QC; ^8^Centre for Outcomes Research and Evaluation, Research Institute of the McGill University Health Centre, Montreal, QC; ^9^Department of Epidemiology, Biostatistics and Occupational Health, School of Population and Global Health, Faculty of Medicine and Health Sciences, McGill University, Montreal, QC; ^10^Science for All Audiences, Toronto, ON; ^11^Department of Psychology, Faculty of Arts, University of Manitoba, Winnipeg, MB; ^12^College of Nursing, Rady Faculty of Health Sciences, University of Manitoba, Winnipeg, MB; ^13^Department of Food and Human Nutritional Sciences, Faculty of Agricultural and Food Sciences, University of Manitoba, Winnipeg, MB

##### **Correspondence:** Ayel Luis R. Batac

**Allergy, Asthma & Clinical Immunology** 2024, **20(Suppl 2)**:33

**Background:** Despite the clear evidence on vaccine safety and efficacy of COVID-19 vaccines, misinformation remains rampant across online platforms. In addition to the dissemination of misinformation online, documented reports of allergic reactions to COVID-19 vaccines have contributed to decreased vaccine confidence among the general population. We aimed to conduct a living scoping review on peer-reviewed and grey literature on COVID-19 vaccine hesitancy and allergic reactions.

**Methods:** Guided by Arksey and O’Malley’s framework for methodological reviews, we searched four scientific databases (MEDLINE, Embase, PsycINFO, CINAHL) using a search strategy developed by content and methodological experts. No restrictions were applied on the type of COVID-19 vaccine, country of study, and language of publication. Grey literature searches were restricted to 10 languages. The study population included patients of all ages. The search was reported according to the Preferred Reporting Items for Systematic reviews and Meta-Analyses extension for Scoping Reviews.

**Results:** Of the 1114 unique records retrieved from our comprehensive search, a total of 54 (4.8%) studies were included. Findings from the current review provide evidence of low prevalence rates of allergic reactions in the general population. Thirteen of the 54 (24%) included studies highlighted vaccine hesitancy due to possibility of an allergic reaction. Additionally, the present review also highlights research on incidents/details of vaccine-related allergic reactions and risk assessment studies of possible allergic reaction to the COVID-19 vaccine. COVID-19 vaccine acceptance among individuals living with allergy and those with no previous known allergic disease history, is influenced by the fear of an allergic reaction.

**Conclusions:** Despite the low prevalence rates of serious vaccine-related adverse reactions, fear of allergic reactions to the COVID-19 vaccine remain to be one of the leading causes of vaccine hesitancy. Therefore, it is imperative to dispel misinformation and ensure that the appropriate information is promoted correctly and widely to reduce vaccine hesitancy.

## 35 Improving access to pediatric allergy care through use of mixed virtual and in-person outreach clinics in northern British Columbia

### Sean Duke^2^, Alanna Escott^3^, Zaneta Lim^2^, Bahar Torabi^2^, Stephanie C. Erdle^2^, Victoria E. Cook^1,3^

#### ^1^University of British Columbia, Victoria, BC, ^2^University of British Columbia, Vancouver, BC, ^3^Community Allergy Clinic, Victoria, BC

##### **Correspondence:** Sean Duke

**Allergy, Asthma & Clinical Immunology** 2024, **20(Suppl 2)**:35

**Background:** Patients living in remote and rural regions of British Columbia (BC) have limited access to pediatric allergy care, including important diagnostic and treatment modalities. Patients have historically travelled to BC Children’s Hospital in Vancouver, BC, to be seen by a pediatric allergist, which is a significant economic burden to families. Outreach clinics, where specialists visit rural communities, and virtual allergy visits, more widely used in the era of COVID-19, are two methods that can improve access to care. Here we describe our experience with the first 1.5 years of a mixed virtual and in-person allergy outreach clinic in Terrace, British Columbia.

**Methods:** In January 2021, we began accepting referrals for patients ages 0–17 years with allergic concerns residing in Northern, BC. Patients were triaged and assigned either an initial virtual or in-person visit, depending on the reason for referral. Follow-up in-person visits were conducted if needed. Two in-person outreach clinics (5 full clinic days) in Terrace, BC, were conducted between March 2021 and April 2022.

**Results:** From January 2021 to April 2022, 55 patients were referred for assessment. Reasons for referral were food allergy (65%), asthma/allergic rhinitis (13%), eczema (7%), drug allergy (7%), hives (5%), and venom allergy (2%). Thirty-eight patients were assigned to an initial virtual visit; of these, 26 required additional visits (1 virtual visit, 27 in person visits). During in-person clinics, we completed a total of 15 oral food challenges, and 4 drug allergy assessments, 3 of which underwent challenges. Seven patients were started on oral immunotherapy (initial doses in outreach clinics, subsequent dose escalations observed virtually).

**Conclusions:** The use of a mixed virtual and in-person outreach clinic model facilitated timely access to pediatric allergy care, including diagnostic and treatment interventions that require in-person management, without the need for patient travel.

## 36 An investigation of the relationships between infantile atopic dermatitis and perinatal anxiety

### Michael A. Golding^1,2^, Elissa M. Abrams^1,2,3^, Michelle Halbrich^2^, Elinor Simons^1,2^, Leslie E. Roos^4^, Jill Keddy-Grant^2^, Tom Gerstner^2^, Jo-Anne St-Vincent^5^, Jennifer LP Protudjer^1,2,6^

#### ^1^Children’s Hospital Research Institute of Manitoba, Wininpeg, MB; ^2^Department of Pediatrics and Child Health, University of Manitoba, Winnipeg, MB; ^3^Department of Pediatrics, University of British Columbia, Vancouver, BC; ^4^Department of Psychology, University of Manitoba, Winnipeg, MB; ^5^The Children’s Allergy and Asthma Education Centre, Winnipeg, MB; ^6^Department of Food and Human Nutritional Sciences, University of Manitoba, Winnipeg, MB

##### **Correspondence:** Michael A. Golding

**Allergy, Asthma & Clinical Immunology** 2024, **20(Suppl 2)**:36

**Background:** Caring for a child with atopic dermatitis (AD) can impose a considerable burden on parents through its impact on infant sleep and its time consuming treatments. While some research has linked this burden to greater levels of psychosocial dysfunction, few studies have investigated the impact of the condition on perinatal anxiety. Consequently, the current study explored whether levels of perinatal anxiety differ between mothers of infants with AD and those without.

**Methods:** The current study recruited mothers with an infant, 18 months or younger, from allergy and dermatology clinics, to complete several self-report measures of physical and psychosocial health. As part of this package, participants completed the Patient-Oriented Scoring Atopic Dermatitis (PO-SCORAD), a measure of AD severity. Multiple regression analyses were used to assess whether mothers with a child with AD reported greater levels of perinatal anxiety relative to the mothers of children without the condition.

**Results:** The final sample was comprised of 33 mothers with an infant with AD and 65 mothers without. The participants had an average age of approximately 31 years (SD = 4.32), while the average infant age was roughly eight months old (SD = 4.25). Mothers with an infant with AD did not significantly differ from controls in their level of perinatal anxiety; however, AD severity was found to predict higher total perinatal anxiety scores (b = 0.34; 95%CI = 0.02; 0.67) as well as greater scores on the subscales related to worry and specific fears (b = 0.16; 95%CI = 0.04; 0.29) and perfectionism, control, and trauma (b = 0.10; 95%CI = 0.002; 0.20).

**Conclusions:** While differences in anxiety between cases and controls failed to reach statistical significance, findings suggest that anxiety does increase with the severity of a child’s AD. More research is needed to determine whether mothers of children with more severe cases of atopic dermatitis are at increased risk of problematic anxiety during the postpartum period.

## 37 Symptoms reported in environmental exposure chamber and symptoms reported during natural season are highly correlated in subjects who reported at least 3 h outdoors during the grass pollen season

### Anne M. Salapatek, Yasmeen Goyal, Patricia Couroux

#### Cliantha Research, Mississauga, ON

##### **Correspondence:** Yasmeen Goyal

**Allergy, Asthma & Clinical Immunology** 2024, **20(Suppl 2)**:37

**Background:** Environmental exposure chambers (EECs) are valuable clinical tools. They provide a controlled yet naturalistic environment to study allergen-induced symptoms. The current study compared the symptoms reported in EEC to those symptoms in natural pollen season.

**Methods:** This study consisted of 7 scheduled visits to the study site, including a medical screening visit; Four EEC visits in which Total Symptom Score (TSS (24) = Total Nasal Symptom Score (12,TNSS) + Total Non-Nasal Symptom Score (12,TNNSS)) were collected over 3 h with grass pollen exposure (3500 ± 500 pollen grains/m^3^); and 2 site visits flanking an at-home assessment of TSS during peak grass season. Subjects also reported the time spent outdoors (6 days). Symptoms were reported on the same electronic patient data acquisition device. Average TSS collected over the EEC sessions and peak grass allergy season (2015) by same subjects were compared. Pearson correlation analyses were performed.

**Results:** The average TSS for subjects in the EEC and during a 6-day period in the field was less variable in EEC than in field**.** Subjects who reported spending > 3 h outdoors had higher average TSS than subjects < 3 h. A low correlation (non-significant) was observed between reported TSS in the EEC and TSS for subjects who spent < 3 h outdoors (r = 0.2131). There was a strong and highly significant correlation between TSS in the EEC and TSS for subjects spending > 3 h outdoors (r = 0.51, p < 0.0001).

**Conclusions:** There is a high correlation between each subject’s response in the EEC and their response in the Field for those who reported at least 3 h outdoors. Conversely, those subjects who spent less time outdoors did not develop adequate symptoms and did not correlate with their response in the EEC. This further supports the use of the EEC to ensure patients are included in allergy trials who have an adequate level of symptoms to test allergy treatments.

## 38 Reduction of anosmia in patients with severe chronic rhinosinusitis with nasal polyps (CRSwNP) treated with dupilumab

### Andrew P. Lane^1^, Joaquim Mullol^2^, Claire Hopkins^3^, Wytske J. Fokkens^4^, Stella E. Lee^5^, Asif H. Khan^6^, Jérôme Msihid^7^, Scott Nash^8^, Radhika Nair^9^, Siddhesh Kamat^8^, Paul J. Rowe^10^, Yamo Deniz^8^, Juby A. Jacob-Nara^10^

#### ^1^Department of Otolaryngology – Head and Neck Surgery, Johns Hopkins University School of Medicine, Baltimore, MD, USA; ^2^Rhinology Unit & Smell Clinic, ENT Department, Hospital Clínic, IDIBAPS, Universitat de Barcelona, CIBERES, Barcelona, Spain; ^3^Department of Otorhinolaryngology, King’s College, London, United Kingdom, ^4^Department of Otorhinolaryngology, Amsterdam University Medical Centres, Amsterdam, Netherlands; ^5^Division of Otolaryngology – Head & Neck Surgery, Brigham and Women’s Hospital, Harvard Medical School, Boston, MA, USA; ^6^Global Medical Affairs, Sanofi, Chilly-Mazarin, France; ^7^Health Economics and Value Assessment, Sanofi, Chilly-Mazarin, France, ^8^Medical Affairs, Regeneron Pharmaceuticals, Inc., Tarrytown, NY, USA; ^9^Health Economics and Value Assessment, Sanofi, Cambridge, MA, USA; ^10^Global Medical Affairs, Sanofi, Bridgewater, NJ, USA

##### **Correspondence:** Andrew P. Lane

**Allergy, Asthma & Clinical Immunology** 2024, **20(Suppl 2)**:38

**Background:** Dupilumab improved sense of smell in patients with CRSwNP versus placebo in the SINUS-24/-52 (NCT02912468/NCT02898454) studies. We report a post hoc analysis of olfactory and clinical outcomes in patients from the SINUS studies.

**Methods:** Patients with baseline smell impairment (loss of smell score ≥ 1 [at least “mild”] and University of Pennsylvania Smell Identification Test [UPSIT, 0–40] ≤ 34/ ≤ 33 [women/men] and at least “very mild problem” in the 22-item Sinonasal Outcome Test [SNOT-22] smell/taste item) who received dupilumab 300 mg or placebo q2w were analyzed. Change from baseline in nasal polyp score (NPS, 0–8)/nasal congestion (NC, 0–3)/SNOT-22 (0–110) was reported for patients achieving normosmia (UPSIT > 34/ > 33 [women/men]) and those reporting anosmia (UPSIT 0–18) at Week 24 (pooled SINUS-24/-52) and 52 (SINUS-52).

**Results:** Most patients had anosmia at baseline (dupilumab n = 322/398 [81%]/placebo n = 213/267 [80%]); 6% in each group had mild hyposmia (UPSIT ≥ 31/30- ≤ 34/33 [women/men]). Among patients with baseline anosmia, the proportions achieving normosmia or mild hyposmia with dupilumab/placebo were 29%/0.5% at Week 24 and 29%/0% at Week 52; 94% of patients remained anosmic at Week 52 with placebo versus 33% with dupilumab. In patients with baseline smell impairment, mean [SD] changes from baseline in NPS/NC/SNOT-22 with dupilumab were greater in patients achieving normosmia than those reporting anosmia at Week 24 (NPS: − 2.56 [1.80] versus − 1.22 [1.65]; NC: − 1.44 [0.70] versus − 1.01 [0.95]; SNOT-22: − 33.96 [20.03] versus − 20.56 [20.50]) and Week 52 (NPS: -3.57 [1.77] versus − 1.16 [1.79]; NC: − 1.68 [0.59] versus − 0.82 [0.84]; SNOT-22: − 29.73 [11.44] versus − 16.79 [19.15]).

**Conclusions:** Most patients with severe CRSwNP had anosmia, of whom approximately one-third treated with dupilumab improved to normosmia/mild hyposmia at Week 24 and 52, while almost all treated with placebo remained anosmic. Improved clinical outcomes were seen in patients reporting better olfactory outcomes, which may have contributed to their improved olfaction.

## 40 A qualitative investigation into vaccine hesitancy and confidence amongst people managing allergy

### Kaitlyn A. Merrill^1,2^, Ayel Luis R. Batac^1,2^, Nicole Askin^3^, Michael A. Golding^1,2^, Elissa M. Abrams^1,2,4^, Phillipe Bégin^5,6^, Moshe Ben-Shoshan^7,8,9^, Erika Ladouceur^10^, Leslie E. Roos^11^, Vladan Protudjer^12^, Jennifer LP Protudjer^1,2,13^

#### ^1^Department of Pediatrics and Child Health, Max Rady College of Medicine, Rady Faculty of Health Sciences, University of Manitoba, Winnipeg, MB; ^2^Children’s Hospital Research Institute of Manitoba, Winnipeg, MB; ^3^WRHA Virtual Library, University of Manitoba, Winnipeg, MB; ^4^Division of Allergy and Immunology, Department of Pediatrics, Faculty of Medicine, University of British Columbia, Vancouver, BC, ^5^Division of Allergy, Department of Pediatrics, Centre hospitalier universitaire Sainte-Justine (CHU Sainte-Justine), Montreal, QC; ^6^Division of Allergy, Department of Medicine, Centre hospitalier de l’Université de Montréal (CHUM), Montreal, QC; ^7^Division of Pediatric Allergy, Clinical Immunology and Dermatology, Department of Pediatrics, Montreal Children’s Hospital, Montreal, QC; ^8^Centre for Outcomes Research and Evaluation, Research Institute of the McGill University Health Centre, Montreal, Quebec, Canada, Montreal, QC; ^9^Department of Epidemiology, Biostatistics and Occupational Health, School of Population and Global Health, Faculty of Medicine and Health Sciences, McGill University, Montreal, QC; ^10^Science for All Audiences, Toronto, ON; ^11^Department of Psychology, Faculty of Arts, University of Manitoba, Winnipeg, MB; ^12^College of Nursing, Rady Faculty of Health Sciences, University of Manitoba, Winnipeg, MB; ^13^Department of Food and Human Nutritional Sciences, Faculty of Agricultural and Food Sciences, University of Manitoba, Winnipeg, MB

##### **Correspondence:** Kaitlyn A. Merrill

**Allergy, Asthma & Clinical Immunology** 2024, **20(Suppl 2)**:40

**Background:** COVID-19 vaccines are critical to the pandemic response. As such, vaccine hesitancy poses a threat to global health. Despite the rarity of allergic reaction to COVID-19 vaccines, people with allergies may still hold reservations. We aimed to describe the perceptions of COVID-19 vaccines amongst individuals managing allergy.

**Methods:** Semi-structured qualitative interviews regarding COVID-19 vaccines were conducted with two categories of participants (all eligible for vaccination): (1) parents of children with allergies, (2) adults with allergies. Participants were recruited via social media. Transcripts were analysed independently by two researchers utilizing thematic analysis.

**Results:** At this time, eight interviews have been conducted (n = 5 parents of children with allergies; n = 3 adults with allergies). All participants (and eligible children of participants) have been vaccinated, with a range of allergies, including food and drug allergies. Thus far, three major themes have been identified: (1) limited resources available regarding allergies and COVID-19 vaccines caused distrust, and ultimately delay, in vaccination, (2) specific allergic disease history not reflected in clinical trials or published literature increased vaccine hesitancy, and (3) allergists’ advice enhanced confidence in obtaining the vaccine. Many participants cited medical professionals, government, and researchers as reputable sources to obtain information surrounding vaccines, despite significant misinformation in the media. Allergy community groups, such as Facebook groups for parents with food allergies, served as social supports and influenced the decision making of others in similar positions.

**Conclusions:** Despite evidence of the safety of COVID-19 vaccines for those with allergies, vaccine confidence was initially shaken due to gaps in resources, professional medical advice, and representation in the literature. Ultimately, the decision to be vaccinated often was influenced by diverse stakeholders, including medical professionals, scientists, and governmental organizations, as well as community groups. Knowledge translation efforts should address the identified gaps to reduce the spread of misinformation.

## 41 Safety of Covid-19 vaccines and effect of Covid-19 infection in patients with mastocytosis

### Alex Nguyen^1^, Catherine K. Zhu^1^, Emilie Groulx-Boivin^1^, Connor Prosty^1^, Sofianne Gabrielli^1^, Vera Laboccetta^2^, Pasquale Mulé^3^, Michelle Le^4^, Elena Netchiporouk^4^, Xun Zhang^2^, Reman Hak^5^, Greg Shand^2^, Sharon Baum^5^, Shoshana Greenberger^6^, Moshe Ben-Shoshan^3^

#### ^1^Faculty of Medicine, McGill University, Montreal, QC; ^2^Research Institute of the McGill University Health Centre, Montreal, QC; ^3^Division of Pediatric Allergy, Immunology, and Dermatology, Montreal Children’s Hospital, Montreal, QC; ^4^Division of Dermatology, McGill University Health Centre, Montreal, QC; ^5^Department of Dermatology, Chaim Sheba Medical Center, Sackler School of Medicine, Tel-Aviv University, Ramat Gan, Israel, ^6^Department of Dermatology, Sheba Medical Center, Ramat Gan, Israel

##### **Correspondence:** Alex Nguyen

**Allergy, Asthma & Clinical Immunology** 2024, **20(Suppl 2)**:41

**Background:** Mastocytosis is characterized by abnormal clonal mast cell proliferation. Given the paucity of data and concern of anaphylaxis in patients with mastocytosis, it is crucial to assess the safety of COVID-19 vaccines in this population. We aimed to assess the risk of allergic reactions and the effect of COVID-19 infection and vaccines among patients with mastocytosis.

**Methods:** Participants were recruited from the Mastocytosis Registry (MASTER; Canada) and Sheba Medical Center (Israel) between December 2021 to May 2022. Consenting participants were administered standardized questionnaires querying: whether they were infected with COVID-19 and reported symptoms, if they received the first and second dose vaccines, and post-vaccination side effects including allergic reactions (urticaria/angioedema, current rash flaring and/or need for updosing medications, or respiratory symptoms) and common side effects including injection site pain and flu-like symptoms.

**Results:** Forty-one participants with mastocytosis were administered a standardized questionnaire (mean age = 19, SD = 20.85; 58.5% male; 57.7% cutaneous mastocytosis; 38.5% systemic mastocytosis; 11.5% mast cell activation syndrome). Amongst all participants, 39.0% reported COVID-19 infection. Most (75.0%) reported flu-like symptoms, 18.7% were asymptomatic, and 6.2% had anosmia/ageusia. Of the 25 participants who were eligible for vaccination (^3^;5 years-old), 84.0% received a first-dose vaccine (71.4% Pfizer, 19.0% Moderna, 9.5% AstraZeneca) and 72.0% received a second-dose vaccine (83.0% Pfizer, 16.7% Moderna). Of those who received the first-dose vaccine, 38.1% reported common symptoms (flu-like symptoms, injection site pain), 14.0% allergic reactions (4.8% current hives flaring, 4.8% angioedema, 4.8% respiratory symptoms, 9.5% chest pain/palpitations, 9.5% ostealgia, and 4.8% diarrhea). Of those who received the second-dose vaccine, 50% reported common symptoms, 5.6% respiratory symptoms, 5.6% chest pain/palpitations, and 5.6% diarrhea. No significant difference was found between side effects of both vaccine doses. No reactions fulfilled the criteria for anaphylaxis in either dose.

**Conclusions:** Our findings suggest that COVID-19 infection and vaccines are well-tolerated in most patients with mastocytosis.

## 42 Mimicker of cellulitis: colophony induced allergic contact dermatitis

### Vivian Szeto^1^, Madeleine Smee^2^, Gordon Sussman^2^

#### ^1^Western University, London, ON, 2. University of Toronto, Toronto, ON

##### **Correspondence:** Vivian Szeto

**Allergy, Asthma & Clinical Immunology** 2024, **20(Suppl 2)**:42

**Background:** Colophony is a plant resin that is found in over 300 different types of products. It is a known allergen that causes bronchial asthma in those exposed to colophony fumes and allergic contact dermatitis in those who use band-aids, anti-wart agents and musicians who play string instruments. Allergic contact dermatitis is also a known mimicker of cellulitis. Differentiating features between the two include: increased rubor and heat in cellulitis, while in allergic contact dermatitis there is more pruritus, presents with tiny vesicles, weeping, fissuring, and crusting of the skin. In this case report, we present a patient with an erythematous lesion with overlying bullae that was initially treated as cellulitis.

**Methods:** Written informed consent was obtained from the patient for the publication of these details.

**Results:** 67-year-old male was stung by an unidentified insect in the posterior aspect of his upper left leg. He was treated with Polypsorin and applied a band aid over the site for a week and a half. He subsequently developed erythema and bullae that spread to the medial and anterior aspect of his left leg. He was assessed at a walk-in-clinic and diagnosed with cellulitis. He was treated with oral cephalexin and transitioned to IV ceftriaxone due to lack of improvement. Patch skin test was performed to (1) paper tape (2) colophony (3) Shoppers Drug Mart band Aid (4) neomycin sulfate (5) Bacitracin. He tested positive to colophony and negative to the other agents with appropriate responses to positive and negative controls.

**Conclusions:** In this case, the patient has been counselled on different products that contain colophony and to avoid them moving forward. This case also highlights the importance of recognizing colophony allergy to increase early removal of the allergen. As well, it reviews the different clinical features between allergic contact dermatitis and colophony.

## 44 Population, delivery and efficacy of patient education in atopic dermatitis: a scoping review

### Bethany F. Wilken, Yuka Asai, Michele Zaman

#### Queen’s University, Kingston, ON

##### **Correspondence:** Bethany F. Wilken

**Allergy, Asthma & Clinical Immunology** 2024, **20(Suppl 2)**:44

**Background:** Atopic dermatitis (AD) is a chronic inflammatory skin disease that affects children and adults. Poor treatment adherence in AD requires interventions to promote self-management; patient education in chronic diseases is key to self-management. Many international AD management guidelines published to date include a recommendation for educating patients as part of their treatment but there are no formal recommendations on how to deliver this knowledge.

**Methods:** A scoping review was performed in the online databases MEDLINE and Ovid in October 2021. The search strategy yielded 388 articles. Of the 388 articles screened, 15 studies met the eligibility criteria, and the quantitative data was summarized by narrative synthesis.

**Results:** The majority of studies were randomized controlled trials conducted in Europe, Asia and North America. Since 2002, there have been limited studies evaluating patient education in the treatment of AD. Frequent education methods used included group-based educational programs, educational pamphlets, individual consultations and online resources. Education was most commonly directed at caregivers and their children. Only one study compared the efficacy of different education methods. In all included studies, the heterogenous nature of outcome measures and study design limited the consistency of results. Despite the heterogeneity of studies, patient education was shown to improve quality of life (QoL), disease severity and psychological outcomes in AD patients.

**Conclusions:** This scoping review highlights that patient education is effective in a variety of domains relevant to AD treatment. Further comparative studies and randomized trials with longer-term follow-up are needed to provide validated and consistent patient education recommendations for AD; these may depend on age and population.

## 45 Real-world impact of cannabis knowledge and attitudes on practice in CSACI members

### Joanna S. Zeiger^1^, William S. Silvers^1,2^, Ajay P. Nayak^3^, David R. Naimi^4^, Isabel J. Skypala^5^, Anne K. Ellis^6^, Lori Connors^7^, Samira Jeimy^8^, Jonathan A. Bernstein^9^, Robert S. Zeiger^1,10^

#### ^1^Canna Research Foundation, Boulder, CO, USA; ^2^University of Colorado School of Medicine, Denver, CO, USA; ^3^Thomas Jefferson University, Philadelphia, PA, USA; ^4^Naval Hospital Camp Pendleton, Oceanside, CA, USA; ^5^Royal Brompton & Harefield Hospitals, Guy’s and St. Thomas’ NHS Foundation Trus, London, United Kingdom; ^6^Queen’s University Department of Medicine, Kingston, ON, ^7^Dalhousie University Department of Medicine, Halifax, NS; ^8^Division of Clinical Immunology and Allergy, Department of Medicine, Western University, London, ON; ^9^University of Cincinnati College of Medicine, Bernstein Allergy Group, Cincinnati, OH, USA; ^10^Department of Clinical Science, Kaiser Permanente Bernard J. Tyson School of Medicine, Pasadena, CA, USA

##### **Correspondence:** Joanna S. Zeiger

**Allergy, Asthma & Clinical Immunology** 2024, **20(Suppl 2)**:45

**Background:** Increasing cannabis consumption in Canada is bound to challenge the practicing allergist with a potential upsurge of cannabis allergy (CA) and exacerbation of asthma symptoms from inhalation. This study aimed to determine the knowledge, attitudes, and real-world practice (KAP) regarding cannabis amongst allergists.

**Methods:** The International Allergist Canna KAP study surveyed 445 members from the Canadian Society of Allergy & Clinical Immunology (CSACI, N = 47), European Academy of Allergy & Clinical Immunology (EAACI, N = 191), and American College of Allergy, Asthma, & Immunology (ACAAI, N = 207). Survey questions included: 13 on cannabis attitudes, 7 on cannabis knowledge, and 4 on practice. SPSS Two-Step cluster analysis grouped participants by attitudes. Chi-square measured differences between the societies on attitudes’ clusters, knowledge score [mean(SD)], and practice.

**Results:** Three attitudes’ clusters were identified: Traditional, Progressive, and Unsure. CSACI members had the most Progressive attitudes (CSACI:66%, EAACI:31%, ACAAI:60%; p < 0.001). ACAAI members had the most knowledge [3.5(1.6)] compared to CSACI [3.3(1.6)] and EACCI [2.4.1.6] (p < 0.001). There were no significant differences between societies in comfort talking to patients about cannabis. However, there were differences in counseling patients who inhale cannabis on cessation [% always: CSACI:(17.0%), EAACI:(35.1%), ACAAI:(30.4%); % never: CSACI:(23.4%), EAACI:(4.7%), ACAAI:(7.7%)] (p < 0.001); asking how often patients use cannabis [% yes: CSACI:(76.6%), EAACI (55.5%), ACAAI:(57.5%)] (p < 0.05); and querying patients about how they use cannabis [% yes: CSACI:(74.5%), EAACI:(40.8%), ACAAI:(59.4%)] (p < 0.001). Suspected CA has been seen by 42.9% of CSACI members and 15.2% have seen patients with asthma exacerbation(s) from cannabis use.

**Conclusions:** Knowledge, attitudes, and real-world patient interactions regarding practice differ by geographical region. A majority of CSACI members asked patients about cannabis use. Education for allergists about cannabis is imperative to best identify and support patients at risk from inhalation methods of cannabis and suspected cannabis allergy.

This abstract is dedicated to Dr. William Silvers.

## 46 Allergies and mental health diagnosis in Canadian primary care settings: exploring the burden of illness

### Alexander Singer^1^, Elissa M. Abrams^1,2^, Leanne Kosowan^1^, Jennifer LP Protudjer^1,3^

#### ^1^Rady Faculty of Health Sciences, University of Manitoba, Winnipeg, MB; ^2^Department of Pediatrics, Division of Allergy and Immunology, University of British Columbia, Vancouver, BC; ^3^Centre for Allergy Research, Karolinska Institute, Stockholm, Sweden

##### **Correspondence:** Alexander Singer

*Allergy, Asthma & Clinical Immunology* 2024, **20(Suppl 2)**:46

**Background:** Comorbid chronic disease and/or adverse social determinants of health are associated with the prevalence and morbidity of allergic diseases. This study will explore associations between mental health conditions including ADHD, mood disorders, eating disorders, anxiety, and depression with atopic diseases.

**Methods:** The Canadian Primary Care Sentinel Surveillance Network (CPCSSN) is the pan-Canadian network that cleans and maintains longitudinal de-identified electronic medical record patient data from 1,574 consenting primary care providers representing 1,493,516 patients in seven Canadian provinces. The CPCSSN allergy table has been categorized to identify the following allergy categories: drug allergy, environment allergy, food allergy, stinging insect allergy, and vaccine allergy. We will estimate the prevalence of mental health disorders amongst patients diagnosed with allergies, by age group and sex, and by province. We will describe the population with and without an allergy using frequency, mean and standard deviation. We will assess associations between allergy and mental health diagnosis using chi-square.

**Results:** There were 308,955 patients with a documented allergy in the CPCSSN allergy table. The most common allergy documented in CPCSSN is drug allergy (39.0%, n = 209,028), environmental allergy (11.0%, n = 62,164) and food allergy (8.0%, 48,822). We will apply validated case definitions for ADHD, mood disorders, eating disorders, anxiety and depression to explore associations between allergies and mental health diagnosis.

**Conclusions:** This work will contribute to the understanding of associations of various allergies and mental health diagnosis in a pan-Canadian primary care dataset. The association between allergies and mental health have long-term implications for patients and the health system. This research will aid in informing strategies to address this problem and further understand the burden of illness related to allergies.

## Case reports

## 48 Chronic new onset psoriasiform eruption after Covid-19 vaccination: a case study

### Khaldon Abbas^1^, Madeleine Smee^2^, Gordon Sussman^3^

#### ^1^Faculty of Medicine, University of British Columbia, Vancouver, BC; ^2^Gordon Sussman Clinical Research Inc, Toronto, ON; ^3^Division of Allergy/Clinical Immunology, University of Toronto, Toronto, ON

##### **Correspondence:** Khaldon Abbas

**Allergy, Asthma & Clinical Immunology** 2024, **20(Suppl 2)**:48

**Background:** Psoriasis is a chronic immune-mediated inflammatory skin condition that may be precipitated by infection, stress, trauma, and vaccination. Psoriasis has been documented in inactivated, attenuated, toxoid, and polysaccharide types of vaccines including influenza, Bacillus Calmette-Guerin, tetanus-diphtheria, and pneumococcal vaccines. Recently, COVID-19 vaccinations have been linked to the exacerbation of psoriasis. We present a case of persistent severe new-onset psoriasiform eruption in a patient following administration of the Pfizer-BioNTech mRNA vaccine.

**Case presentation:** A previously healthy 66-year-old female presents with a psoriasiform eruption 7 days following an initial single dose of Pfizer-BioNTech mRNA vaccine. The patient had no past history of psoriasis, eczema, urticaria, angioedema, or anaphylaxis, and no family history of immune dysfunction. The psoriasiform eruption was severe upon onset and it was generalized itchy and scaly. There were excoriations present on the buttock, trunk, and scalp. She did not have any previous COVID-19 infection. She was initially treated with high dose antihistamines, 40 mg cetirizine per day, and topical steroids, betamethasone, but they were not effective in managing her symptoms. She later decided to see a naturopath and she was given red clover and milk thistle bark herbal medicines and probiotics which were also ineffective. The itchy and scaly eruptions and excoriations persisted for 8 months before slowly starting to resolve. One year after psoriasiform eruption onset, there still persists some mild excoriations on the buttocks and trunk area.

**Conclusions:** This case highlights a lesser-known reaction to the Pfizer-BioNTech mRNA vaccine. There is no well-understood pathologic mechanism for new-onset or flares of psoriasis following vaccination. To the best of our knowledge, this is the first case of chronic severe psoriasiform eruption due to the Pfizer-BioNTech mRNA vaccine that persisted for over 8 months in a patient with no history of psoriasis.

**Statement of consent**

Written Informed Consent for this case report was obtained from the patient.

## 49 Idiopathic hypereosinophilic syndrome successfully treated with mepolizumab: a case report

### Jonathan Keow^1^, Kamilia Rizkalla^1^, Kara Robertson^2^

#### ^1^Department of Pathology and Laboratory Medicine, Western University, London, ON; ^2^Division of Clinical Immunology and Allergy, Department of Medicine, Western University, London, ON

##### **Correspondence:** Khaldon Abbas

**Allergy, Asthma & Clinical Immunology** 2024, **20(Suppl 2)**:49

**Background:** Idiopathic hypereosinophilic syndrome (HES) is defined as eosinophilia associated with organ involvement in the absence of a secondary cause or a recurrent genetic abnormality. Recently, mepolizumab has been approved in Canada as the only targeted biologic therapeutic for the treatment of adult patients with idiopathic HES.

**Case presentation:** A 20-year-old otherwise healthy Caucasian female with no recent travel or occupational exposures was referred with an 18-month history of rhinitis, dyspnea, and exercise intolerance. Spirometry demonstrated an FEV1 of 83% with borderline obstruction. There was serum eosinophilia with an increase in absolute eosinophil count (AEC) 1.5 × 10^9^/L to 4.6 × 10^9^/L over 1 month.

A workup of secondary causes was negative. Vitamin B12, C-reactive protein, serum tryptase, immunoglobulin levels, antinuclear antibody levels and antineutrophil cytoplasmic antibody levels were normal. Serology for HIV and strongyloides was negative. There was no biochemical evidence of adrenal insufficiency (normal AM cortisol). Her kidney and liver function were normal, and no organomegaly was identified with computed tomography scans of the thorax and abdomen. However, an echocardiogram revealed pericarditis necessitating a referral to Cardiology for assessment of end-organ dysfunction.

She was also referred to Hematology for bone marrow biopsy, during which her AEC rose to 12.2 × 10^9^/L. Her marrow showed marked eosinophilia. No *PDGFRA* or *PDGFRB* rearrangement and no clonal abnormalities were detected. She failed treatment with high-dose prednisone and methotrexate, and is now on mepolizumab with improvement.

**Conclusions:** Morbidity and mortality from HES results from tissue infiltration by eosinophils and subsequent organ dysfunction from eosinophil degranulation. The relationship between eosinophilia duration and severity is not well established, but an AEC greater than 1.5 × 10^9^/L has been recommended as a threshold for starting treatment. Mepolizumab should be considered as a treatment option in patients who fail first-line therapy with glucocorticoids.

**Statement of consent**

Written Informed Consent for this case report was obtained from the patient

## 50 Choriocarcinoma in a 37-year old pregnant patient taking omalizumab

### Michelle Kwok, Geneviève Genest

#### McGill University, Montreal, QC

##### **Correspondence:** Michelle Kwok

**Allergy, Asthma & Clinical Immunology** 2024, **20(Suppl 2)**:50

**Background:** Atopy is a state of general immune hyperresponsiveness and IgE may be involved in cancer immunosurveillance. Higher serum IgE levels correlate with a decreased malignancy risk and prolonged survival in cancer patients. Conversely, IgE deficiency is associated with an increased risk of cancer, such as breast and ovary.

Omalizumab is an anti-IgE monoclonal antibody indicated for chronic spontaneous urticaria (CSU) and atopic asthma. While a theoretical cancer risk has been suspected in patients receiving Omalizumab data from post-marketing studies and systematic reviews have not substantiated this.

**Case presentation:** We present a G1P1 37-year-old female with severe, refractory CSU and angioedema. Her quality of life drastically improved after starting omalizumab. The patient desired another pregnancy, and after failed attempts at discontinuing Omalizumab, a decision was made to conceive while on treatment. Her second pregnancy was a blighted ovum, abortion was induced at 8 weeks gestation. Her third pregnancy was an invasive mole that transformed into choriocarcinoma requiring surgical removal and chemotherapy.

A pedigree revealed strong family history of malignancy, particularly her mother who had choriocarcinoma at 49 and breast cancer at age 51. Genetic work-up for hereditary cancer syndromes did not reveal any pathogenic variants. Given the refractory nature of her urticaria, a decision was made to continue the Omalizumab with increased cancer surveillance.

**Conclusions:** This patient likely developed choriocarcinoma because of a cancer predisposition syndrome. However, contribution of Omalizumab cannot be excluded. Some limitations of pre-existing studies on Omalizumab and malignancy include selection bias, lack of pre-enrolment data, and short observation period. A recent disproportionality analysis suggested that omalizumab may be associated with increased cancer risk. Discussing a theoretical yet clinically unquantifiable and unsubstantiated neoplastic risk with all patients prior to stating Omalizumab is unjustified and likely harmful. However, disclosing this possibility may be appropriate for certain patients to ensure shared decision making.

**Statement of Consent**

Written Informed Consent for this case report was obtained from the patient.

## 51 Elevated tryptase level in a child with idiopathic anaphylaxis: a case of hereditary alpha tryptasemia

### Vicky LeBlanc^1,2^, Wade Watson^1,2^

#### ^1^Dalhousie University, Halifax, NS; ^2^IWK Health Centre, Halifax, NS

##### **Correspondence:** Vicky LeBlanc

*Allergy, Asthma & Clinical Immunology* 2024, **20(Suppl 2)**:51

**Background:** Hereditary alpha-tryptasemia (HaT), caused by increased copies of *TPSAB1*, is a autosomal dominant disorder affecting approximately 5% of the general population. Elevated baseline tryptase level is a consistent finding among affected families. However, clinical features differ greatly among individuals. While some are asymptomatic, others complain of functional gastrointestinal symptoms, recurrent cutaneous manifestations or constitutional symptoms. HaT has also been associated with increased severity of venom anaphylaxis, systemic mastocytosis (12%) and idiopathic anaphylaxis (17%). Uncertainty remains around the clinical phenotype of HaT.

**Case presentation:** We describe a 5 year 8 month girl with 2 episodes of idiopathic anaphylaxis manifesting by urticaria, angioedema, pruritus and emesis. First episode occurred after swimming for one hour and the second, 48 h after an acute febrile illness. The second episode required 2 doses of epinephrine for symptoms to resolve. Despite a detailed history, no allergic trigger could be identified. The tryptase level during the second episode was elevated at 31.2 mg/L. Baseline tryptase levels were measured twice at 22.7 and 20.6 mg/L. She is otherwise healthy, non-atopic child, with no other complaints. A meticulous physical examination revealed no sign of lymphadenopathy or hepatosplenomegaly. Only one small hyperpigmented area was identified on her left leg, but Darier’s sign was negative. Investigations included normal complete blood count, normal liver and renal functions and negative genetic testing for KIT mutation. Baseline serum tryptase levels were measured in both parents. Her mother had a normal level of 7 mg/L and her father had an elevated level of 17 mg/L. He has no symptoms.

**Conclusions:** In conclusion, HaT is a condition with a great spectrum of clinical manifestations. While idiopathic anaphylaxis is not a common presentation, it should be considered in patients presenting with recurrent idiopathic anaphylaxis.

**Statement of consent**

Written Informed Consent for this case report was obtained from the patient’s guardian.

## 52 Anaphylaxis to ground flaxseed in a pediatric patient tolerant to whole flaxseed: case report

### Kevin K. Lee, Stephanie Erdle

#### The University of British Columbia, Vancouver, BC

##### Correspondence*:* Kevin K. Lee

**Allergy, Asthma & Clinical Immunology** 2024, **20(Suppl 2)**:52

**Background:** The benefits of flaxseed (linseed) as a source of fiber and omega-3 fatty acids makes it a popular superfood that has garnered interest as an egg-substitute when prepared by mixing ground flaxseed with water. The increased prevalence of flaxseed in the diet has led to concerns that flaxseed is an emerging allergen. Although sensitization and anaphylaxis to flaxseed have previously been reported, we report the first case of anaphylaxis to ground flaxseed used as an egg-substitute in a pediatric patient who regularly consumes whole flaxseed. Written informed consent for publication was obtained.

**Case presentation:** A 10-month-old girl with atopic dermatitis was assessed at the BC Children’s Hospital Allergy Clinic after she developed lethargy, projectile vomiting, and generalized urticaria within 15 min of consuming a small bite of vegan muffin. Symptoms spontaneously resolved within an hour. She had no further exposure to the muffin nor any anaphylactic events.

Potential allergenic foods in the muffin included walnut and flaxseed, the latter of which was ground and baked into the muffin as an egg-substitute. As she tolerated whole flaxseeds several times per week but had not yet introduced tree nuts to her diet, walnut was suspected to be the allergen. However, skin prick test (SPT), serum specific IgE, and oral food challenge to walnut were negative. Instead, flaxseed allergy was diagnosed with a positive SPT of 12 × 6 mm and serum specific IgE of 51.1 kU/L; its avoidance was recommended.

**Conclusions:** Whole flaxseed may be tolerated in patients allergic to ground flaxseed due to its hard outer coat which prevents it from being digested and exposing the allergen; similar findings were noted in sesame-allergic patients. Inquiry of ground or whole flaxseed consumption during food allergy assessments and careful review of ingredients in foods made with substitutions are advised.

## 53 Development of IgE-mediated reaction to egg after its avoidance for food protein-induced enterocolitis syndrome: case report

### Kevin K. Lee, Stephanie Erdle

#### The University of British Columbia, Vancouver, BC

##### Correspondence*:* Kevin K. Lee

**Allergy, Asthma & Clinical Immunology** 2024, **20(Suppl 2)**:53

**Background:** The avoidance of causative agent in food protein-induced enterocolitis syndrome (FPIES) management is discordant to the recommendation of early and regular consumption of allergenic foods to prevent food allergies. Consequently, IgE-mediated reactions have occurred after FPIES for cow’s milk. Reports of such reactions with eggs are limited. Our case adds to this limited literature along with an atypical worsening of FPIES reaction over time. Written informed consent for publication was obtained.

**Case presentation:** A 10-month-old girl was diagnosed with FPIES to egg with negative skin prick test (SPT) at the BC Children’s Hospital Allergy Clinic after two episodes of recurrent projectile vomiting, pallor, and non-bloody diarrhea occurring 3 to 4 h after egg consumption at ages 7 and 8 months, requiring intravenous rehydration and antiemetics. She was reassessed at 21 months with an oral food challenge (OFC) to scrambled egg. She developed a single vomiting episode at 4 h, which was treated with sublingual ondansetron and oral rehydration. Skin testing to egg was repeated prior to FPIES challenge at 37 months given her history of atopic dermatitis. This revealed new sensitization to egg white (5 × 4 mm) and yolk (3 × 3 mm). French toast containing 1 egg was given in incremental doses for OFC given her sensitization. A crumb-sized portion was tolerated. 10 min after consuming 1/16th of the French toast, she developed 3 to 4 urticaria on her face and back which resolved in 20 min. After 3 h, she developed significant projectile vomiting, pallor, and lethargy requiring transfer to the emergency room for intravenous rehydration and antiemetics.

**Conclusions:** New IgE-mediated reaction to egg was observed after its avoidance for FPIES, suggesting that SPT prior to OFC in atopic patients and counselling on the risk of developing new IgE-mediated allergy secondary to strict avoidance is prudent.

## 54 An usual presentation of idiopathic hypereosinophilic syndrome with features of eosinophilic granulomatosis with polyangiitis and substantial response to mepolizumab

### Adhora Mir, Timothy Olynych

#### University of Ottawa, Ottawa, ON

##### **Correspondence:** Adhora Mir

**Allergy, Asthma & Clinical Immunology** 2024, **20(Suppl 2)**:54

**Background:** Eosinophilia with multiple organ involvement characterizes a wide yet specific group of atopic, infectious, neoplastic, and immunologic disorders. Eosinophilic granulomatosis with polyangiitis (EGPA) is one such vasculitic disorder with respiratory tract manifestations including asthma and rhinosinusitis. When there is a lack of evidence for other known causes of eosinophilia, the diagnosis of hypereosinophilic syndrome (HES), which describes a rare group of disorders defined by sustained hypereosinophilia over six months and evidence of organ involvement, can be considered. In general the treatment of HES is dependent on etiology, however a novel indication allows Il-5 monoclonal antibody therapy to be used when there is no identifiable non hematologic secondary cause.

**Case presentation:** We describe a case of idiopathic hypereosinophilic disorder in a 24 year old asthmatic male presenting with chronic rhinosinusitis, aphthous stomatitis, eosinophilic cellulitis, gastrointestinal involvement, and elevated IgG4 without identifiable underlying etiology of eosinophilia despite extensive workup. Features of EGPA were predominant however the lack of vasculitic evidence precluded this diagnosis. While Well’s syndrome was proposed to describe his cutaneous manifestations, it was not sufficient to account for his presentation. Our patient achieved remarkable clinical improvement of symptoms and corticosteroid independence with mepolizumab. There was no recurrence of symptoms at 8 month follow-up.

**Conclusions:** While this case behaves like a secondary eosinophilic disease and shares many characteristics with EGPA, the absence of vasculitis and other disease criteria necessitates the diagnosis of idiopathic hypereosinophilic syndrome. Our case contributes to the growing base of literature on the efficacy and long term safety of mepolizumab for idiopathic hypereosinophilic syndrome.

**Statement of consent**

Written Informed Consent for this case report was obtained from the patient.

## 55 Bear meat anaphylaxis: a case report

### Ariana Mustillo^1^, Michael Fein^2^

#### ^1^Centre Hospitalier de l’Université de Montréal, Montreal, QC; ^2^McGill University Health Center, Montreal, QC

##### **Correspondence:** Ariana Mustillo

**Allergy, Asthma & Clinical Immunology** 2024, **20(Suppl 2)**:55

**Background:** Meat allergy is uncommon and bear meat allergy is comparatively exceedingly rare. It has exclusively been reported within the context of a larger family of mammalian meat IgE-mediated allergy involving galactose-a-1,3-galactose(alpha-gal), typically associated with delayed symptom onset after meat ingestion. Here we report the first case to our knowledge of a non-alpha-gal related bear meat allergy.

**Case presentation:** A 46-year-old Cree male with no significant prior medical history, no history of atopy or known allergies presented with a suspected anaphylactic reaction to bear meat. Fifteen minutes after ingestion of boiled black bear meat, the patient developed throat swelling, shortness of breath, nausea, vomiting and weakness. He had no urticaria or angioedema. He was treated in his local community and received two doses of intramuscular epinephrine with complete resolution of symptoms within an hour after treatment. All other persons who consumed the bear meat did not develop any reaction. The patient had since avoided bear meat, but has tolerated every other consumed meat, including beef, rabbit, and moose. He also developed localized leg swelling when he applied bear fat to his skin. The patient was later assessed for skin testing in our allergy clinic. He had a positive skin prick test (SPT) to boiled black bear meat with a 6 mm wheal and a negative saline control. Aeroallergens were also tested by SPT, which were positive for cat (9 mm), dust mites (D. Farinea 8 mm; D. Pteronyssinus 7 mm) and cockroach (7 mm) with no associated symptoms of allergic rhinitis.

**Conclusions:** The rapid symptom onset and tolerance of other mammalian meat rules out alpha-gal as a culprit in this patient’s bear meat allergy. To our knowledge, this is the first report in the literature of an allergy specifically to bear meat and clinicians should be aware of the existence of this entity when evaluating patient’s for meat allergy.

**Statement of consent**

Written Informed Consent for this case report was obtained from the patient.

## 56 Extra-pulmonary manifestations of Covid-19 can occur. A case report of a Covid-19 infection presenting as rhabdomyolysis in Trinidad and Tobago

### Ijaz Ogeer^1^, Meghan Lalla^1^, Joseph Zacherali^1^, Narine Mack^1^, Stanley Giddings^2^

#### ^1^Augustus Long Hospital, Point A Pierre, Trinidad and Tobago; ^2^University Of The West Indies, St. Augustine, Trinidad and Tobago

##### **Correspondence:** Ijaz Ogeer

**Allergy, Asthma & Clinical Immunology** 2024, **20(Suppl 2)**:56

**Background:** Acute respiratory manifestations of COVID-19 infection have been well documented. Other organ systems may also be adversely affected. However, there is a limited understanding of the extent and management of extrapulmonary COVID‐19‐related conditions. Infection-related rhabdomyolysis is rare but may be seen as a manifestation of COVID-19. The case below highlights COVID-19 infection presenting as rhabdomyolysis in Trinidad and Tobago.

**Case presentation:** A 40-year-old male presented with generalized muscle pains for 4 days associated with dark coloured urine and intermittent fever. There were no pulmonary symptoms and he denied any recent strenuous activity or exercise. Past medical history was positive for Hepatitis A. Drug history included simple analgesics. His vaccination status was up to date as per local guidelines. Cardiac and respiratory examinations were normal. Urinalysis was strongly positive for blood. Lab investigations revealed a normal complete blood count, troponin I, renal and hepatic function. Chest radiograph and abdominal ultrasound scans were also normal. PCR for Covid-19 was positive together with a severely elevated creatine kinase (CK) value of 193,792 U/L. A diagnosis of rhabdomyolysis was made, intravenous fluid therapy was initiated and he was transferred to the intensive care unit. Serial labs were performed to monitor CK and renal function. CK trend showed steady decline reaching a value of 2762 U/L on day 8. There was resolution of symptoms and he was discharged to return for outpatient follow up.

**Conclusions:** Non-specific symptoms of myalgia and arthralgia occur often in viral illnesses. In Covid-19 infection these symptoms can occur without respiratory involvement and should herald the screening for rhabdomyolysis to rule out this critical extrapulmonary manifestation.

**Statement of consent**

Written Informed Consent for this case report was obtained from the patient.

## 57 A case of eosinophilic granulomatosis and polyangiitis following Pfizer-BioNTEch COVID-19 mRNA vaccination

### Jane Park, Stephen Betschel

#### Division of Clinical Immunology and Allergy, Department of Medicine, University of Toronto, Toronto, ON

##### **Correspondence:** Jane Park

**Allergy, Asthma & Clinical Immunology** 2024, **20(Suppl 2)**:57

**Background:** The global pandemic with coronavirus disease 19 (COVID-19) continues and massive vaccination programs against COVID-19 have become the mainstay of public health measures. There have been some case reports of ANCA-associated vasculitis (AAV) following COVID-19 vaccinations. It is a rare but important complication that can present similarly to COVID-19 infection. We present the first case in Canada of AAV following COVID-19 vaccination with worsening renal function upon receiving the second dose vaccination.

**Case presentation:** We report a case of a 38-year-old-male who developed respiratory and flu-like symptoms within 3 weeks of receiving his first dose of Pfizer-BioNTech COVID-19 mRNA vaccine. He had persistent progressive symptoms which were initially attributed to possible severe acute respiratory syndrome coronavirus 2 (SARS-CoV-2) infection. This led to a considerable diagnostic delay. He eventually presented to an emergency department approximately 8 weeks after symptom onset. He had pulmonary and renal involvement. Bronchoscopy and renal biopsy ultimately confirmed the diagnosis of proteinase 3 (PR3) antibody, anti-neutrophil cytoplasmic antibody (ANCA)-positive, eosinophilic granulomatosis and polyangiitis (EPGA). Despite being on systemic glucocorticoid therapy, he had worsening renal failure after receiving his second dose of Pfizer-BioNTech COVID-19 mRNA vaccine. The patient had renal recovery after starting rituximab in addition to continuing oral glucocorticoids.

**Conclusions:** As COVID-19 vaccination programs continue for the foreseeable future, there will likely be more cases of AAV following COVID-19 vaccines. While direct causality cannot be proven, our case raises the suspicion with strong temporal association that COVID-19 vaccine may likely have triggered an autoimmune response. We highlight the importance of early diagnostic work-up and considering the diagnosis EGPA when patients present with unexplained pulmonary renal disease following COVID-19 vaccination.

**Statement of consent**

Written Informed Consent for this case report was obtained from the patient.

## 58 Dupilumab induced psoriasis in a patient with atopic dermatitis: a case report

### Madeleine Smee^1^, Khaldon Abbas^2^, Gordon Sussman^3,1^

#### ^1^Gordon Sussman Clinical Research Inc., North York, ON; ^2^Faculty of Medicine, University of British Columbia, Vancouver, BC; ^3^Division of Allergy/Clinical Immunology, University of Toronto, Toronto, ON

##### **Correspondence:** Madeleine Smee

**Allergy, Asthma & Clinical Immunology** 2024, **20(Suppl 2)**:58

**Background:** Atopic dermatitis (AD) is a common skin disease characterized by chronic inflammation and pruritus due to hypersensitivity of skin and mucous membranes. Dupilumab is an IgG4 monoclonal antibody that inhibits interleukin (IL)-4 and IL-13 signalling and is used to treat AD. Common side-effects include injection site reactions, conjunctivitis, and keratitis. Additionally, several cases of psoriasis developing after treatment with dupilumab have been recorded. Psoriasis is characterized by excessive growth of epithelial cells into plaques that constantly shed. We describe a case of new onset psoriasis following dupilumab treatment.

**Case Presentation:** A 55-year-old female was initially seen in 2018 for severe unresponsive AD that covered 50–75% of her skin surface area. She had failed first-, second-, and third-line treatments prior to dupilumab. Failed treatments included antihistamines, topical steroids, and Omalizumab. Dupilumab was initially prescribed for 150 mg every two weeks but frequency was increased to weekly after six months without clinical response. She had significant clinical improvement and her AD became completely controlled, however four years after starting dupilumab she developed psoriasis. Her psoriasis is severe, generalized, and mainly affects the lower limbs and buttocks. It is well-demarcated, patchy, with thick white scale. The patient wished to continue therapy with dupilumab despite side effects because of success in clearing her AD. To date, the psoriasis persists.

**Conclusions:** The patient is under further investigation on how to effectively treat her AD without triggering psoriasis. This case highlights a lesser-known reaction to dupilumab, and promotes caution in the initiation of Dupilumab therapy in patients with AD and concurrent psoriasis.

**Statement of consent**

Written Informed Consent for this case report was obtained from the patient

## 59 Preadolescent with red dragonfruit allergy without previous ingestion history

### Joanne Wang, Godfrey Lam

#### University of British Columbia, Vancouver, BC

##### **Correspondence:** Joanne Wang

**Allergy, Asthma & Clinical Immunology** 2024, **20(Suppl 2)**:59

**Background:** Dragon fruit belongs to the Cactaceae family located in Central America and Asia. Nonspecific lipid transfer proteins (nsLTPs) are ubiquitous in plants and can cause systemic reactions to plant foods in sensitized individuals. Previously, pitaya nsLTP has been identified as an allergen causing anaphylaxis.

**Case presentation:** An 11-year-old female, with history of atopic dermatitis, allergic rhinitis, consumed a red dragonfruit for the first time. She developed periorbital swelling, generalized urticaria, and subjective dyspnea within two hours of ingestion. She did not require epinephrine, and her symptoms improved within 24 h. She saw her general physician, who recommended she avoid dragonfruit, kiwi, and passionfruit. Prior to this, she was tolerating kiwifruit and passionfruit infrequently without reactions. Patient was assessed in the allergy clinic, where she was found to have positive epicutaneous skin tests to grass pollen, dust mite dander, and dragon fruit (fresh food, 6 mm). She was also found to have positive sIgE to kiwifruit (0.66 KU/L) and passionfruit (0.61KU/L). Based on history and skin test results, she has a confirmed dragonfruit allergy. Her low titre sIgE to kiwifruit and passionfruit may be sensitization rather than true allergies. She was offered oral food challenges to these in the future.

**Conclusions:** Dragonfruit allergy is rare. To our knowledge, only three cases have been documented thus far. NsLTP is one of the most frequent causes of primary food allergies given its widespread distribution throughout the plant kingdom in botanically unrelated foods. NsLTP was identified as an allergen in pitaya in one case. Our patient has never ingested dragon fruit, and her sensitization may have occurred via nsLTPs in other plant foods (kiwifruit) or pollens (grass). Her sensitisation to kiwifruit, in particular, is interesting as Zhu et al. previously described a patient with kiwifruit anaphylaxis who was also allergic to dragon fruit suggesting possible shared protein.

**Statement of consent**

Written Informed Consent for this case report was obtained from the patient’s guardian.

## 60 Role of treating allergic rhinitis and asthma in a patient with chronic spontaneous urticaria

### Melanie M. Wong^1^, Paul K. Keith^2^

#### ^1^Michael G. DeGroote School of Medicine, McMaster University, Hamilton, ON; ^2^Department of Medicine, McMaster University, Hamilton, ON

##### **Correspondence:** Melanie M. Wong

**Allergy, Asthma & Clinical Immunology** 2024, **20(Suppl 2)**:60

**Background:** Patients with chronic spontaneous urticaria (CSU) are more likely to have elevated levels of aeroallergen sensitization, blood eosinophil %, total IgE, and IgG autoantibodies to FcεR1α, IgE and thyroid peroxidase, indicating the importance of T2 inflammation in the pathogenesis of CSU.

**Case presentation:** We describe a 44-year-old female with recurrent CSU, allergic rhinitis (AR), intermittent asthma and previous ethmoid sinusitis on CT. In December 2019, she presented to the ED with lip and tongue angioedema and generalized urticaria, with no clear trigger. She received epinephrine intramuscularly and was discharged on prednisone.

An autoimmune screen was positive for autoantibodies to thyroglobulin and dsDNA. Her TSH was normal and C3 was low. Her IgE was 11 kIU/L (normal < 87). Her blood eosinophil count was 0.3 × 10^9^/L (normal < 0.4), but her blood eosinophil percentage was 5% (normal < 4%). Skin prick tests had been positive to grass, ragweed, and dust mite.

This patient was counselled on allergen avoidance and started on intranasal fluticasone furoate and montelukast for her nasal congestion, and rupatadine for her CSU. Her CSU and AR worsened during the 2020 grass and ragweed pollen seasons. During summer 2020, she experienced two CSU flares requiring prednisone. We increased her rupatadine dosage and started inhaled fluticasone furoate to control asthma symptoms. She was offered omalizumab but declined. By 2021, her CSU symptoms markedly improved. She required only one course of prednisone during ragweed season and had better control of her hives in grass season. Since October 2021, she has been off all medications and has not experienced any flares until spring 2022, when she decided to initiate ragweed sublingual immunotherapy.

**Conclusions:** This patient’s urticaria deteriorated seasonally and improved with managing her mucosal allergic inflammation. Specific allergen avoidance and desensitization may be beneficial in the management of CSU.

**Statement of consent**

Written Informed Consent for this case report was obtained from the patient

## 61 Omega 5 gliadin allergy: are there differences in whole wheat vs white flour?

### Kavya Yatham^1^, Juan Ruiz^2^

#### ^1^University of Saskatchewan, Saskatoon, SK; ^2^University of British Columbia, Vancouver, BC

##### **Correspondence:** Kavya Yatham

*Allergy, Asthma & Clinical Immunology* 2024, **20(Suppl 2)**:61

**Background:** Omega 5 gliadin (O5G) allergy is an uncommon allergy to a component of wheat that can result in anaphylaxis, typically in the presence of a cofactor. Making the diagnosis can be difficult, as patients can present various allergic manifestations and may be unknowingly exposed to wheat. The most common cofactors identified are exercise, alcohol, and nonsteroidal anti-inflammatory drugs. A small number of cases have no identifiable cofactor found.

**Case presentation:** We present a case of a 23-year-old female with recurrent hives, GI upset, vomiting, and presyncope to multiple foods over two years. These symptoms all occurred within one hour of ingestion of various foods. She experienced no hives outside of these instances. She had experienced seven episodes of anaphylaxis with no apparent trigger. Skin prick testing was positive to white, milled, and gluten flour; negative to wheat and other grains such as rye, barley, and oat. IgE testing to O5G IgE/Tri a 19 (5.20 kU/L) was positive. She has continued to eat whole wheat products regularly with no anaphylaxis but has reacted to ingesting flour products such as hamburger buns, French baguettes, and pizza. We considered the possibility of flour mite anaphylaxis however it is unlikely that she would react to many different types of flour products and consistently react to white flour products from various sources.

She failed an oral challenge to white flour in the form of baked pizza. Within one hour of ingestion, she developed disseminated hives.

**Conclusions:** We present a case of O5G allergy with multiple episodes of anaphylaxis with no identifiable cofactor. Of particular interest, the patient never reacted to eating whole wheat products. We hypothesize that there may be differences in the processing of whole wheat flour versus white flour and the content of O5G in such products.

**Statement of Consent**

Written Informed Consent for this case report was obtained from the patient.

## Urticaria/angioedema

## 62 HAE with normal C1 esterase inhibitor (HAEnC1INH): treatment and attack frequency changes from 2017 to 2020 based on data from the Canadian National Patient Surveys

### Jacquie M. Badiou^1^, Michelle Cooper^1^, Daphne Dumbrille^1^, Robert Bick^2^, Suzanne M. Kelly^3^, Paul Keith^4^, Amin Kanini^5^, Chrystyna Kalicinsky^6^, William H. Yang^7^

#### ^1^HAE Canada, Ottawa, ON; ^2^Health Policy Consultant, Markham, ON; ^3^Red Maple Trials, Inc., Ottawa, ON; ^4^McMaster, Hamilton, ON; ^5^University of British Columbia, Vancouver, BC; ^6^University of Manitoba, Winnipeg, MB; ^7^Ottawa Allergy Research Corporation, Ottawa, ON

##### **Correspondence:** Jacquie M. Badiou

**Allergy, Asthma & Clinical Immunology** 2024, **20(Suppl 2)**:62

**Background:** Hereditary angioedema with normal C1 esterase inhibitor (HAEnC1INH) is a rare genetic disorder which results in unpredictable attacks of angioedema. There are no approved treatments for HAEnC1INH although there are treatments for HAE with low levels or dysfunctional C1INH. We explored the use and impact of these treatments in patients with HAEnC1INH.

**Methods:** Online surveys were sent to Canadian HAE patients in 2017 and 2020 to better understand treatment and health burden. We extracted the responses of patients who reported having HAEnC1INH to evaluate treatment use and attack frequency. Data was analyzed as the percent of responses to a given question.

**Results:** In 2017, There were 26 respondents (88% female) who self-identified as having HAEnC1INH and 45 (84% female) in 2020. HAE treatments were used by 73% in 2017 and 86% in 2020. C1INH either intravenous (IV) or subcutaneous (SQ) was the most common medication used to treat acute attacks (2017: IV 50%; 2020: IV 30%, SQ 11.4%) and for prophylaxis (2017: IV 27%; 2020: IV 33%, SQ 24%).

The percent of patients with > 12 attacks in the prior year decreased from 85% in 2017 to 50% in 2020. The percent who had no attacks that were untreated rose from 0.0% to 58%. Most patients had no hospital and clinic visits (2017: 39%; 2020: 44%). Emergency room and clinic visits were largely unchanged but fewer patients had frequent (> 7/per year) doctor visits (2017: 43%; 2020: 11%).

**Conclusions:** Patients with HAEnC1INH received treatments approved for HAE with low or dysfunctional C1INH in both 2017 and 2020 and the proportion increased in 2020. There was a parallel decrease in attacks and in untreated attacks.

## 63 Autoimmune progesterone dermatitis with concomitant progesterone induced anaphylaxis

### Vivian Szeto, Jumana Sarraj, Samira Jeimy

#### Western University, London, ON

##### **Correspondence:** Vivian Szeto

*Allergy, Asthma & Clinical Immunology* 2024, **20(Suppl 2)**:63

**Background:** Autoimmune progesterone dermatitis (APD) is a rare cutaneous reaction from the release of endogenous progesterone during the luteal phase of the menstrual cycle. The onset of the symptoms typically occurs 3–10 days prior to menses and resolves within 1–2 days after the end of the menstrual cycle. Progesterone induced anaphylaxis (PIA) is an even rarer entity that can accompany APD. In this situation, patients will present with cutaneous eruptions with one additional organ system involvement, often respiratory symptoms. In this case report, we present a patient with APD and concomitant PIA.

**Methods:** Written informed consent was obtained from the patient for the publication of these details.

**Results:** 50-year-old female presents with a 5-year history of ongoing generalized pruritic and evanescent annular welts. The eruptions occur 2 days prior to the onset of her menstrual cycle and resolves after the end of her menses. She also has concomitant respiratory symptoms which includes dyspnea, cough, nasal congestion and left sided chest pain with her eruptions. A skin prick test to progesterone at a concentration of 50 mg/mL was performed yielding negative results. An intradermal graded progesterone challenge was performed with the following concentrations: 0.1 mg/mL, 0.5 mg/mL, 1 mg/ mL. She tested positive for all three intradermal concentrations confirming her diagnosis of APD with anaphylaxis.

**Conclusions:** Treatment of APD includes antihistamines, suppression of ovulation through oral contraceptive agents or hysterectomy for curative intent. In patients with PIA, carrying an adrenaline kit may also be necessary depending on their treatment choice. In this patient, she had entered menses; therefore, no further treatment was necessary. This case highlights the importance of performing a review of systems in patients with APD to assess for concomitant PIA. As well, the need for further research to standardize the diagnosis of APD and PIA.

**Statement of Consent**

Written Informed Consent for this case report was obtained from the patient.

## 64 Safety of Covid-19 mRNA vaccination and effect of Covid-19 infection in children with chronic urticaria

### Catherine K. Zhu^1^, Alex Nguyen^1^, Connor Prosty^1^, Sofianne Gabrielli^1^, Vera Laboccetta^2^, Greg Shand^2^, Pasquale Mulé^3^, Elena Netchiporouk^4^, Michelle Le^4^, Xun Zhang^2^, Sharon Baum^5^, Reman Hakroush^6^, Shoshana Greenberger^6^, Moshe Ben-Shoshan^3^

#### ^1^McGill University Faculty of Medicine, Montreal, QC; ^2^Research Institute of the McGill University Health Centre, Montreal, QC; ^3^Division of Pediatric Allergy, Immunology, and Dermatology, Montreal Children’s Hospital, Montreal, QC; ^4^Division of Dermatology, McGill University Health Centre, Montreal, QC; ^5^Department of Dermatology, Sheba Medical Center, Sackler School of Medicine, Tel-Aviv University, Ramat Gan, Israel; ^6^Department of Dermatology, Sheba Medical Center, Ramat Gan, Israel

##### **Correspondence:** Catherine K. Zhu

**Allergy, Asthma & Clinical Immunology** 2024, **20(Suppl 2)**:64

**Background:** Given the sparse data on the safety of Coronavirus disease 2019 (COVID-19) vaccines in children with chronic urticaria (CU) and the potential risk of urticaria flare post-vaccination, we aimed to assess the safety of the Pfizer-BioNTech BNT162b2 COVID-19 vaccine and the characteristics of COVID-19 infection among CU children.

**Methods:** This study recruited children with CU (5–18 years old) from Montreal Children’s Hospital, The Children’s Clinic (Canada), and Sheba Medical Center (Israel) between December 2021-March 2022. Participants were administered standardized questionnaires on the first and second vaccines (Pfizer), post-vaccination side effects: allergic reaction (urticaria/angioedema, current CU flares/need for updosing CU medications, respiratory symptoms, anaphylaxis), flu-like symptoms (chills, fatigue, myalgia, low-grade fever), injection site reaction (pain, redness), and high fever (> 39C). Data were also collected on participants infected by COVID-19. Data analysis was performed using R v.4.2.0.

**Results:** A total of 101 vaccine eligible CU children responded to the questionnaire (median age = 13.0 years; 49.5% male; 75.2% chronic spontaneous urticaria; 13.9% chronic inducible urticaria; 10.9% coexistence of both), 73.3% received the first vaccine and 55.4% received the second. All patients denied allergic reaction post-vaccine. Of the patients who received the first vaccine: 8.1% reported flu-like symptoms, 27.0% injection site reaction, and 1.4% high fever. For the second vaccine: 17.9% reported flu-like symptoms, 32.1% injection site reaction, and 1.8% high fever. No significant difference (Chi-squared test) was found between the side effects of the first and second vaccines. Among all children, 16.8% reported COVID-19 infection (52.9% being unvaccinated at the time of infection). Most infected children (58.8%) reported flu-like symptoms, 5.9% anosmia/ageusia, 5.9% hives flare-up, and none reported anaphylaxis.

**Conclusions:** Our findings suggest that Pfizer-BioNTech COVID-19 vaccination is safe among CU children and does not appear to cause adverse events or CU flares. Among our cohort, the majority had mild COVID-19 symptoms and no hives flare-up post-infection.

## 65 Covid-19 affairs and HAE flares: hereditary angioedema attacks with Covid-19 vaccination and infection

### Tess Robart^1^, Gina Lacuesta^2^, Stephen Betschel^3^, Rozita Borici-Mazi^4^, Victoria E. Cook^6^, Chrystyna Kalicinsky^5^, Amin Kanani^7^, Paul Keith^9^, Louis Marois^8^, Susan Waserman^9^

#### ^1^Dalhousie University Department of Pediatrics, Halifax, NS; ^2^Nova Scotia Health, Halifax, NS; ^3^University of Toronto Department of Clinical Immunology and Allergy, Toronto, ON; ^4^Queen’s University Division of Allergy & Immunology, Kingston, ON; ^5^University of Manitoba Department of Allergy and Immunology, Winnipeg, MB; ^6^University of British Columbia Department of Clinical Immunology and Allergy, Victoria, BC; ^7^University British Columbia Division of Allergy and Immunology, Vancouver, BC; ^8^Laval University Department of Allergy and Immunology, Quebec, QC, ^9^McMaster University Division of Clinical Immunology and Allergy, Hamilton, ON

##### **Correspondence:** Tess Robart

**Allergy, Asthma & Clinical Immunology** 2024, **20(Suppl 2)**:65

**Background:** Hereditary angioedema (HAE) is a rare autosomal dominant disease characterized by recurrent attacks of swelling of the skin and mucous membranes. Risk factors for attacks include exercise, stress, infection, and potentially vaccination. As a result, it is possible that both infection with Covid-19 and vaccination may precipitate attacks of HAE, resulting in increasing needs for on demand and preventative therapy to control the attacks. The effect of Covid-19 vaccination on the HAE attacks was reported during the Covid-19 pandemic, though no data is available in Canada. This study aims to explore the relationship between Covid-19 vaccination and infection, and the frequency and severity of hereditary angioedema attacks in individuals with HAE in Canada.

**Methods:** We will employ an online survey through the ‘Opinio’ survey platform to gather self-reported data around the rate of Covid-19 vaccination, infection, HAE attacks and need for HAE treatment in HAE patients. This survey will be shared with HAE patients from the Hereditary Angioedema Canada (HAEC) network and the Angio-oedeme Hereditaire du Quebec (AOHQ) patient network. Data will undergo descriptive statistical analysis for presentation. The primary outcome measure is the rate and severity of HAE attacks associated with Covid-19 vaccination and infection. Secondary outcome measures will assess the severity of Covid-19 infection amongst HAE patients. The results will help guide treatment for patients with HAE, providing insight to the impact of Covid-19 vaccination and infection, the potential need for risk reduction of HAE attacks with prophylactic therapy, and associated morbidity and mortality.

**Results:** Results from analysis of the descriptive data will be helpful in understanding the effects of Covid-19 vaccination and infection in Canada.

**Conclusions:** Understanding the effects of Covid-19 vaccination and infection in HAE patients may be important in planning for managing treatment in future.

